# Promotion of Expansion and Differentiation of Hematopoietic Stem Cells by Interleukin-27 into Myeloid Progenitors to Control Infection in Emergency Myelopoiesis

**DOI:** 10.1371/journal.ppat.1005507

**Published:** 2016-03-18

**Authors:** Jun-ichi Furusawa, Izuru Mizoguchi, Yukino Chiba, Masayuki Hisada, Fumie Kobayashi, Hiroki Yoshida, Susumu Nakae, Akihiko Tsuchida, Tetsuya Matsumoto, Hideo Ema, Junichiro Mizuguchi, Takayuki Yoshimoto

**Affiliations:** 1 Department of Immunoregulation, Institute of Medical Science, Tokyo Medical University, Tokyo, Japan; 2 Department of Gastrointestinal and Pediatric Surgery, Tokyo Medical University, Tokyo, Japan; 3 Department of Infectious Diseases, Kyorin University of Medicine, Tokyo, Japan; 4 Department of Biomolecular Sciences, Faculty of Medicine, Saga University, Saga, Japan; 5 Laboratory of Systems Biology, Center for Experimental Medicine and Systems Biology, Institute of Medical Science, University of Tokyo, Tokyo, Japan; 6 Department of Microbiology, Tokyo Medical University, Tokyo, Japan; 7 Department of Cell Differentiation, The Sakaguchi Laboratory of Developmental Biology, Keio University School of Medicine, Tokyo, Japan; 8 Department of Immunology, Tokyo Medical University, Tokyo, Japan; McGill University, CANADA

## Abstract

Emergency myelopoiesis is inflammation-induced hematopoiesis to replenish myeloid cells in the periphery, which is critical to control the infection with pathogens. Previously, pro-inflammatory cytokines such as interferon (IFN)-α and IFN-γ were demonstrated to play a critical role in the expansion of hematopoietic stem cells (HSCs) and myeloid progenitors, leading to production of mature myeloid cells, although their inhibitory effects on hematopoiesis were also reported. Therefore, the molecular mechanism of emergency myelopoiesis during infection remains incompletely understood. Here, we clarify that one of the interleukin (IL)-6/IL-12 family cytokines, IL-27, plays an important role in the emergency myelopoiesis. Among various types of hematopoietic cells in bone marrow, IL-27 predominantly and continuously promoted the expansion of only Lineage^−^Sca-1^+^c-Kit^+^ (LSK) cells, especially long-term repopulating HSCs and myeloid-restricted progenitor cells with long-term repopulating activity, and the differentiation into myeloid progenitors in synergy with stem cell factor. These progenitors expressed myeloid transcription factors such as *Spi1*, *Gfi1*, and *Cebpa/b* through activation of signal transducer and activator of transcription 1 and 3, and had enhanced potential to differentiate into migratory dendritic cells (DCs), neutrophils, and mast cells, and less so into macrophages, and basophils, but not into plasmacytoid DCs, conventional DCs, T cells, and B cells. Among various cytokines, IL-27 in synergy with the stem cell factor had the strongest ability to augment the expansion of LSK cells and their differentiation into myeloid progenitors retaining the LSK phenotype over a long period of time. The experiments using mice deficient for one of IL-27 receptor subunits, WSX-1, and IFN-γ revealed that the blood stage of malaria infection enhanced IL-27 expression through IFN-γ production, and the IL-27 then promoted the expansion of LSK cells, differentiating and mobilizing them into spleen, resulting in enhanced production of neutrophils to control the infection. Thus, IL-27 is one of the limited unique cytokines directly acting on HSCs to promote differentiation into myeloid progenitors during emergency myelopoiesis.

## Author Summary

Emergency myelopoiesis is inflammation-induced hematopoiesis that is critical for controlling infection with pathogens, but the molecular mechanism remains incompletely understood. Here, we clarify that one of the interleukin (IL)-6/IL-12 family cytokines, IL-27, plays an important role in emergency myelopoiesis. Among various types of hematopoietic cells in bone marrow, IL-27 predominantly and continuously promoted expansion of only Lineage^−^Sca-1^+^c-Kit^+^ (LSK) cells, especially long-term repopulating hematopoietic stem cells, and differentiation into myeloid progenitors in synergy with stem cell factor. These progenitors expressed myeloid transcription factors such as *Spi1*, *Gfi1*, and *Cebpa/b* through activation of signal transducer and activator of transcription 1 and 3, and had enhanced potential to differentiate into neutrophils, but not into plasmacytoid dendritic cells. Among various cytokines, IL-27 in synergy with stem cell factor had the strongest ability to augment the expansion of LSK cells and their differentiation into myeloid progenitors. The blood stage of malaria infection was revealed to enhance IL-27 expression through interferon-γ production, and IL-27 then promoted the expansion of LSK cells, differentiating and mobilizing them into the spleen, resulting in enhanced production of neutrophils to control the infection. Thus, IL-27 is one of the limited unique cytokines directly acting on hematopoietic stem cells to promote differentiation into myeloid progenitors during emergency myelopoiesis.

## Introduction

Emergency myelopoiesis is inflammation-induced hematopoiesis, which is critical for controlling systemic infection with pathogens such as a virus, bacteria, or parasite [1,2]. In contrast to adaptive immune cells such as T cells and B cells, which can vigorously proliferate in response to their specific antigens, innate immune cells need to be replenished from hematopoietic stem cells (HSCs) and progenitors in bone marrow (BM) because of their low proliferative activity. However, the molecular mechanism of emergency myelopoiesis during infection remains incompletely understood. HSCs and hematopoietic progenitors can directly sense the presence of pathogens via pattern recognition receptors (Rs) such as Toll-like receptors (TLRs), and they can also respond to pro-inflammatory cytokines such as interferon (IFN)-α, IFN-γ, interleukin (IL)-1, tumor necrosis factor (TNF)-α, and granulocyte colony-stimulating factor (G-CSF) [1]. IFN-α and IFN-γ have pleiotropic effects on many cell types, including HSCs and hematopoietic progenitors [1]. Recently, these cytokines were demonstrated to induce an expansion of HSCs and myeloid progenitors, leading to the production of mature myeloid cells [3–6], although their inhibitory effects on hematopoiesis were previously reported [7–9]. Currently, thus, there are several conflicting positive and negative effects of IFN-α and IFN-γ in hematopoiesis [10,11]. However, these discrepancies may be explained by compensatory mechanisms, including IFN-γ-mediated secretion of other cytokines such as IL-6 [12] and fms-related tyrosine kinase 3 ligand (Flt3L) [13].

IL-27 is one of the IL-6/IL-12 family cytokines; it plays important roles in immune regulation with both pro-inflammatory and anti-inflammatory properties [14–16]. IL-27 consists of p28 and Epstein-Barr virus-induced gene 3 (EBI3), and its receptor is composed of WSX-1 and glycoprotein (gp)130, which is a common receptor subunit in many of the IL-6 family cytokines. We previously demonstrated that IL-27 plays a role in HSC regulation, and that IL-27 expands HSCs and promotes their differentiation *in vitro* [17]. Moreover, transgenic (Tg) mice expressing IL-27 showed enhanced myelopoiesis in BM and extramedullary hematopoiesis in the spleen [17].

In the present study, we further examined the effects of IL-27 on hematopoiesis, the molecular mechanisms, and the physiological role of IL-27 in the control of malaria infection. IL-27 acted on and expanded Lineage (Lin)^−^Sca-1^+^c-Kit^+^ (LSK) cells, which are highly enriched in HSCs together with very primitive hematopoietic progenitors [18,19], in BM cells in synergy with stem cell factor (SCF, c-Kit ligand) and differentiated HSCs into myeloid progenitors through activation of signal transducer and activator of transcription 1 (STAT1) and STAT3. Moreover, malaria infection induced IFN-γ production, which augmented IL-27 expression, and the IL-27 then promoted the expansion and mobilization of LSK cells into the spleen, resulting in enhanced myelopoiesis to resolve the infection. Our results revealed that IL-27 is one of the limited unique cytokines directly acting on long-term HSCs (LT-HSC), which represent the true stem cells capable of self-renewing, and promotes the expansion and differentiation of them into myeloid progenitors.

## Results

### IL-27 and SCF expand only LSK cells among various kinds of BM progenitors

**Fig 1:**
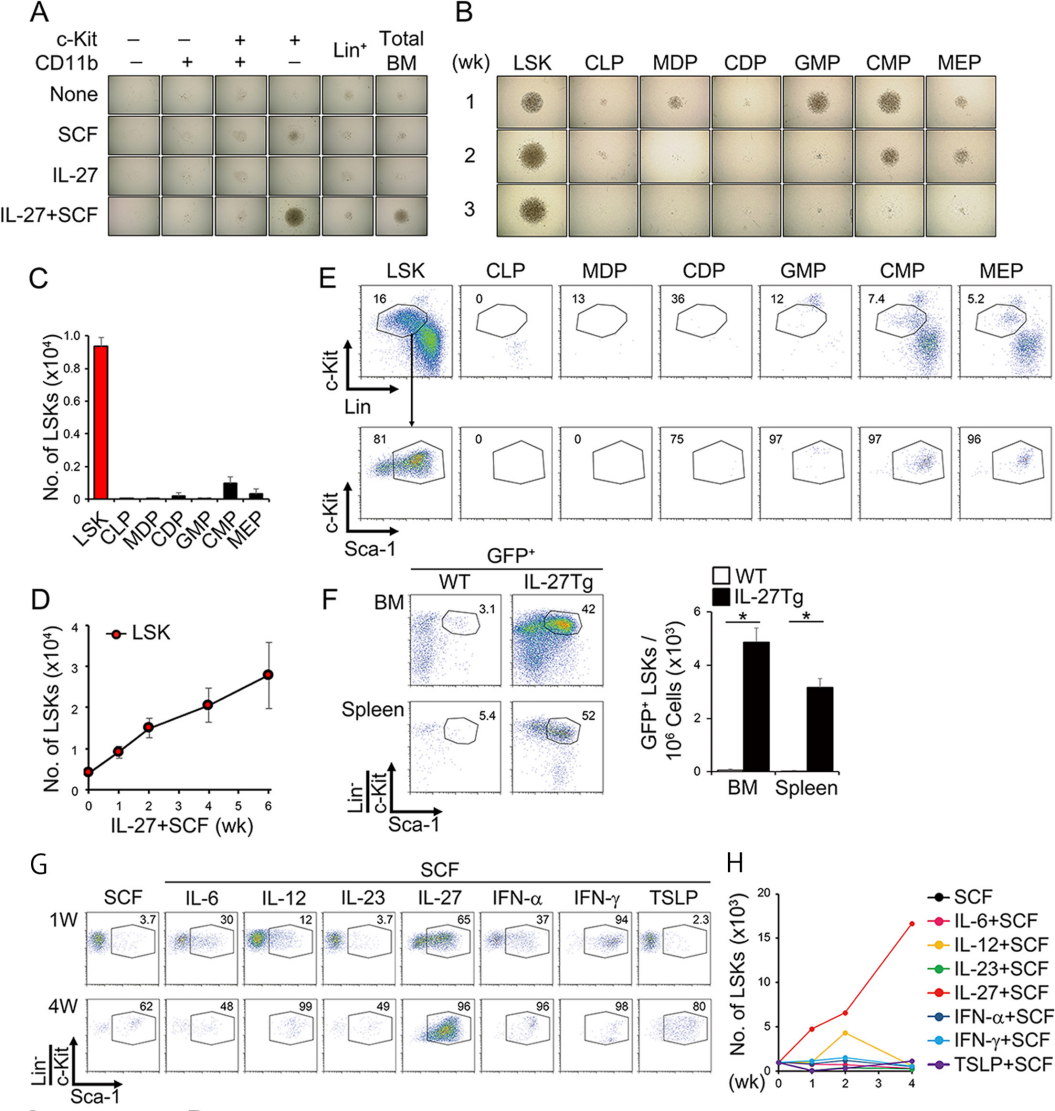
IL-27 and SCF most strongly expand only LSK cells among various kinds of BM progenitors and differentiate them into myeloid progenitors retaining the LSK phenotype. (A) Expansion of only the c-Kit^+^CD11b^−^Lin^−^ population by IL-27 and SCF in BM cells. Total BM cells were divided into two populations positive or negative for Lin markers, and the Lin^−^ population was further divided into four populations positive for either c-Kit or CD11b, both positive, or both negative. Each population (5 × 10^3^) purified by sorting was stimulated by either IL-27 or SCF alone, or both, in a round 96-well plate and photographed 2 weeks later. (B-E) Expansion of only the LSK cell population by IL-27 and SCF among various BM progenitors. BM progenitors (4–5 × 10^3^) purified by sorting were stimulated with IL-27 and SCF. Cell expansion was photographed at indicated periods (B) and cell number of the expanded LSK cell population retaining the LSK phenotype was counted at 1 week (C). Time kinetic analysis of cell numbers in the LSK population retaining the LSK phenotype (D). Representative flow cytometry dot plot analysis of c-Kit and Lin (upper) and of c-Kit and Sca-1 in the Lin^−^c-Kit^+^ population (lower) of expanded LSK cells and progenitor cells at 1 week (E). (F) Expansion of LSK populations *in vivo* by IL-27 in *IL-27* Tg mice. LSK cells were purified by sorting from BM cells of *GFP* Tg mice and transferred into non-lethally irradiated WT and *IL-27* Tg mice. Twenty days later, BM and spleen in these recipient mice were analyzed for GFP^+^ LSK populations. Data are shown as mean ± SEM (n = 2–3) and are representative of at least two independent experiments. *P < 0.05. (G-H) Augmented and prolonged expansion of the LSK cell population by only IL-27 and SCF *in vitro*. LSK cells (1 × 10^3^) from WT mice were stimulated by various cytokines together with SCF for 1 to 4 weeks, the stimulated cells were analyzed by flow cytometry (G), and cell number was counted with time course (H). Data are representative of at least two independent experiments.

Previously, we demonstrated that stimulation of LSK cells with IL-27 and SCF induces an expansion of HSCs and hematopoietic progenitors, including short-term repopulating cells [17]. Moreover, we found that only the combination of IL-27 and SCF, but not either alone, vigorously and continuously expands BM cells to produce LSK cells and CD11b^+^c-Kit^−^ cells [17]. To examine which cell populations in BM cells respond to IL-27 and SCF in more detail, BM cells were divided into two populations positive or negative for Lin markers except CD11b, and the Lin^−^ population was further divided into four populations positive for either c-Kit or CD11b, or both positive, or both negative. Each population purified by sorting was then stimulated with IL-27 and SCF. Among these five populations, only the Lin^−^c-Kit^+^ population greatly expanded (Fig 1A). Next, the BM cells were divided into respective hematopoietic progenitors according to the expression of cell surface markers, as reported previously [20–22], and stimulated with IL-27 and SCF. Only the LSK cell population vigorously and continuously expanded over more than 6 weeks, although transient and slight expansion was seen in the cell populations of granulocyte/macrophage progenitor (GMP), common myeloid progenitor (CMP), and megakaryocyte/erythrocyte progenitor (MEP) (Fig 1B–1E). The expanding cells in the LSK cell population were further analyzed for the expression of cell surface markers. In line with the preliminary results, there seemed to be two populations, the phenotypical LSK population and the Lin^+^c-Kit^−^ population (Fig 1E).

We previously demonstrated that *IL-27* Tg mice, which express high amounts of IL-27 in blood, show an increased number of LSK cells in the BM and spleen [17]. To further examine the *in vivo* effects of IL-27 on the expansion of LSK cells, LSK cells were purified by sorting from BM cells of *GFP* Tg mice and transferred into wild-type (WT) and *IL-27* Tg mice. The transferred GFP^+^ LSK cells vigorously expanded in the BM and spleen of *IL-27* Tg mice, but not in those of WT mice, and approximately half of the expanding cells retained the cell surface markers for LSK phenotype (Fig 1F). These results suggest that IL-27 vigorously and continuously augments the expansion of LSK cells both *in vitro* and *in vivo*.

### IL-27 has the strongest ability to augment the expansion of LSK cells and their differentiation into myeloid progenitors retaining the LSK phenotype

Because it was previously reported that IFN-α and IFN-γ induce proliferation of HSCs *in vivo* [3–5], we next explored the effects of various cytokines in collaboration with SCF on the expansion of LSK cells *in vitro*. However, IFN-α and IFN-γ augmented the expansion of LSK cells very little, and only IL-27 enhanced it vigorously over 4 weeks (Fig 1G and 1H). Moreover, although there are several cytokines, such as IL-3, IL-11, G-CSF, and TPO, that are known to transiently expand and differentiate HSCs [1], none showed an ability superior to that of IL-27 in expanding LSK cells retaining the LSK phenotype over a long period of time (S1 Fig). Thus, IL-27 has the strongest ability to augment the expansion of LSK cells.

### LSK cells expanded by IL-27 and SCF are multipotent myeloid progenitors

**Fig 2:**
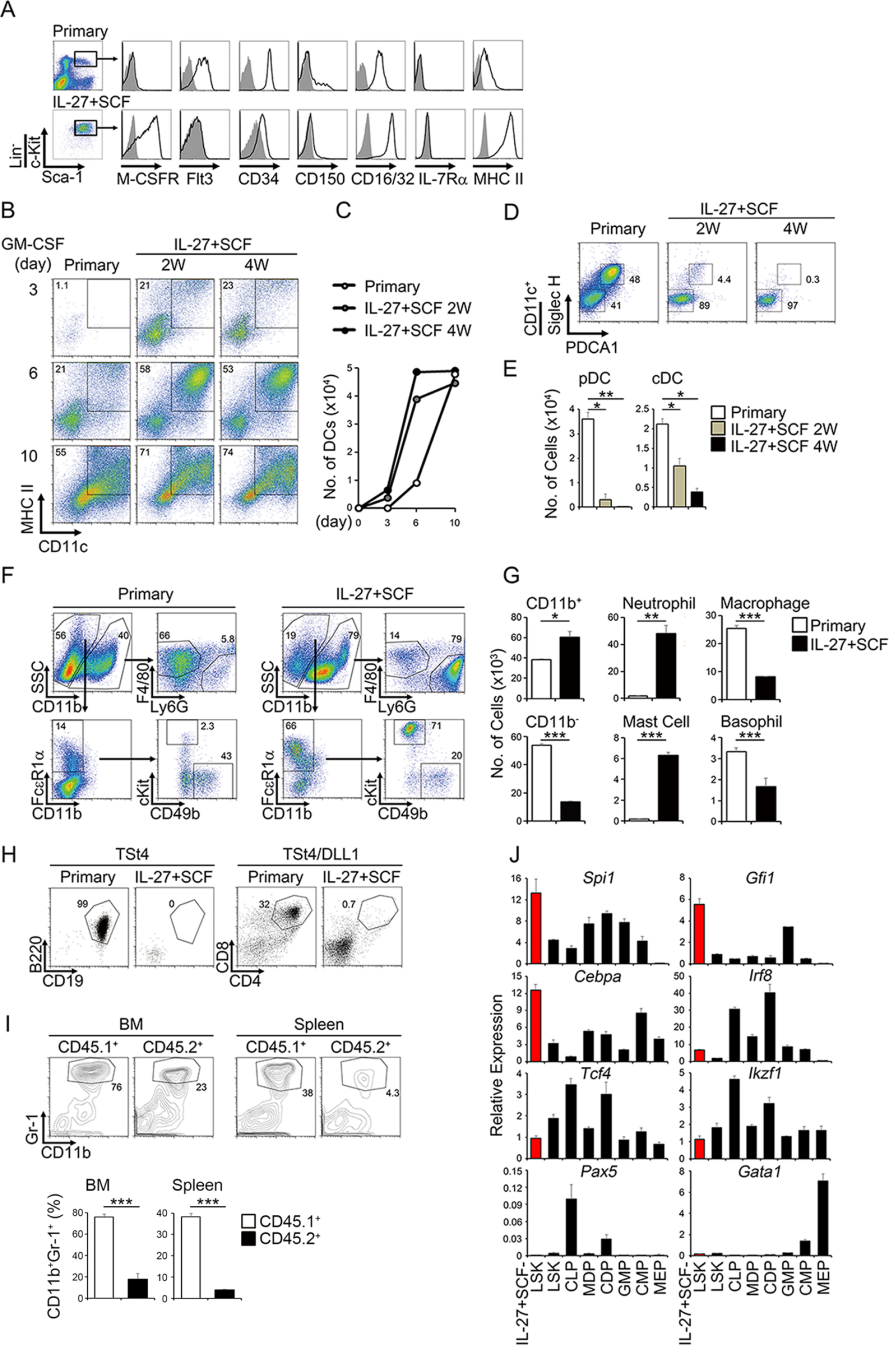
LSK cells expanded by IL-27 and SCF are multipotent myeloid Progenitors. (A) Flow cytometry histogram analysis of cell surface markers in the LSK cells expanded by IL-27 and SCF for 4 weeks and primary BM cells using antibodies as indicated (solid line) and their control antibodies (plane line with shading). (B-C) Augmented potential of the LSK cells expanded by IL-27 and SCF to differentiate into mDCs. LSK populations (3 × 10^3^) purified from the LSK cells expanded by IL-27 and SCF for 2 and 4 weeks and primary BM cells were stimulated with GM-CSF. After the indicated time, these stimulated cells were analyzed for the expression of MHC class II and CD11c (B), and the cell numbers of mDC (MHC class II^+^CD11c^+^) were counted (C). (D-E) Decreased potential of the LSK cells expanded by IL-27 and SCF to differentiate into pDCs (Siglec H^+^PDCA1^+^CD11c^+^) and cDCs (Siglec H^−^PDCA1^−^CD11c^+^). (F-G) Enhanced potential of the LSK cells expanded by IL-27 and SCF for 2 weeks to differentiate into neutrophils. Neutrophil; Ly6G^+^CD11b^+^, macrophage; F4/80^+^CD11b^+^, mast cell; c-Kit^+^FcεR1α^+^CD11b^−^, basophil; CD49b^+^FcεR1α^+^CD11b^−^. (H) Abrogated potential of the LSK cells expanded by IL-27 and SCF for 2 weeks to differentiate into T (day 18) and B (day 15) cells. (I) Enhanced potential of the LSK cells expanded by IL-27 and SCF to differentiate into neutrophils *in vivo*. LSK populations purified from the LSK cells from CD45.1 congenic mice expanded by IL-27 and SCF for 2 weeks were transferred into sublethally irradiated CD45.2 recipient mice with the same congenic BM cells. Development of Gr-1^+^CD11b^+^ neutrophils in the BM and spleen were analyzed by flow cytometry after 9 days, and the percentage of neutrophils in each CD45.1^+^ and CD45.2^+^ cells was compared. (J) Increased expression of transcription factors critical for the differentiation into myeloid cells in the LSK cells expanded by IL-27 and SCF. RNA was prepared from the LSK population purified from LSK cells expanded for 2 weeks together with other progenitors and subjected to real-time RT-PCR. Data are shown as mean ± SEM (n = 2–4) and are representative of two to three independent experiments. *P < 0.05, **P < 0.01, ***P < 0.005.

The LSK cells expanded by IL-27 and SCF were further analyzed for the cell surface expression of various markers, and the expression levels were compared with those of primary LSK cells freshly prepared from BM of WT mice. The expression levels of macrophage colony-stimulating factor receptor (M-CSFR), CD16/32, and MHC class II in the LSK cells expanded by IL-27 and SCF were much higher than those in primary LSK cells (Fig 2A). The expression levels of CD34 and CD150 in the expanded LSK cells were slightly less than those in primary LSK cells (Fig 2A). In contrast, the expanded LSK cells were almost completely negative for Flt3 expression, whereas primary LSK cells were positive for Flt3 (Fig 2A). Thus, IL-27 and SCF expand and differentiate primary LSK cells into M-CSFR^+^Flt3^−^CD16/32^+^ LSK cells (myeloid progenitor cells).

Next, multipotency of the LSK cells expanded by IL-27 and SCF were examined under various differentiation conditions for migratory dendritic cells (mDCs) by granulocyte/macrophage (GM)-CSF, plasmacytoid DCs (pDCs) and conventional DCs (cDCs; lymphoid-resident DCs) were examined using Flt3L and thrombopoietin (TPO) [23], myeloid cells were examined using IL-3 and SCF, and T cells and B cells were examined by using thymic stromal cells (TSt4) with and without expressing Notch ligand Delta-like 1 (DLL1), respectively [24]. The expanded LSK cells much more rapidly proliferated and differentiated into MHC class II^+^CD11c^+^ mDCs than primary LSK cells in response to GM-CSF, although the total number of mDCs achieved seemed to be similar for both (Fig 2B and 2C). However, LSK cells stimulated with IL-27 and SCF rapidly lost the ability to differentiate into pDC and cDC (Fig 2D and 2E). These phenomena are highly consistent with the almost complete abolishment of Flt3 expression on the expanded LSK cells (Fig 2A). Under myeloid differentiation conditions, the expanded LSK cells differentiated much more greatly into neutrophils and slightly into mast cells, but less so into macrophages and basophils (Fig 2F and 2G). Similar enhanced differentiation into myeloid cells was observed using the LSK cells obtained from WT and *IL-27* Tg mice (S2 Fig). In contrast, the ability to differentiate into B cells and T cells was almost completely abrogated in the expanded LSK cells (Fig 2H).

Moreover, the ability of the LSK cells expanded by IL-27 and SCF to differentiate into myeloid cells *in vivo* was explored by using mixed BM chimeras. The equal cell numbers of the LSK cell population expanded from CD45.1 congenic mice and BM cells from CD45.2 congenic mice were mixed and transferred into sublethally irradiated CD45.2 congenic mice. After 9 days, cell populations of neutrophils in the BM and spleen were analyzed. In agreement with the *in vitro* results described, the percentages of neutrophils derived from the expanded LSK cells were markedly higher than those from primary LSK cells in both BM and spleen (Fig 2I).

To explore the molecular mechanisms whereby IL-27 and SCF expand LSK cells, total RNA was prepared from the expanded LSK cells and respective hematopoietic progenitors and analyzed with real-time reverse transcriptase-polymerase chain reaction (RT-PCR). The expanded cells highly expressed the transcription factors critical for differentiation into myeloid cells such as *Spi1*, *Gfi1*, and *Cebpa*, but expression was much less for those important for the other types of cells such as *Irf8*, *Tcf4*, and *Ikzf1* [25–27] (Fig 2J). No expression of transcription factors important for B cells and erythrocytes, *Pax5* [28] and *Gata1* [29], respectively, was observed. In addition, the expression of *Cbepb*, which is a transcription factor recently demonstrated to be regulated by cytokines and control emergency granulopoiesis [30–32], was also increased (S3 Fig).

These results suggest that LSK cells expanded by IL-27 and SCF are multipotent myeloid progenitors that have unique potential to differentiate into mDCs, neutrophils, and mast cells, and less so into macrophages, and basophils, but not into pDCs, cDCs, T cells, and B cells.

### STAT1 and STAT3 are important for expansion and differentiation of LSK cells by IL-27 and SCF

**Fig 3:**
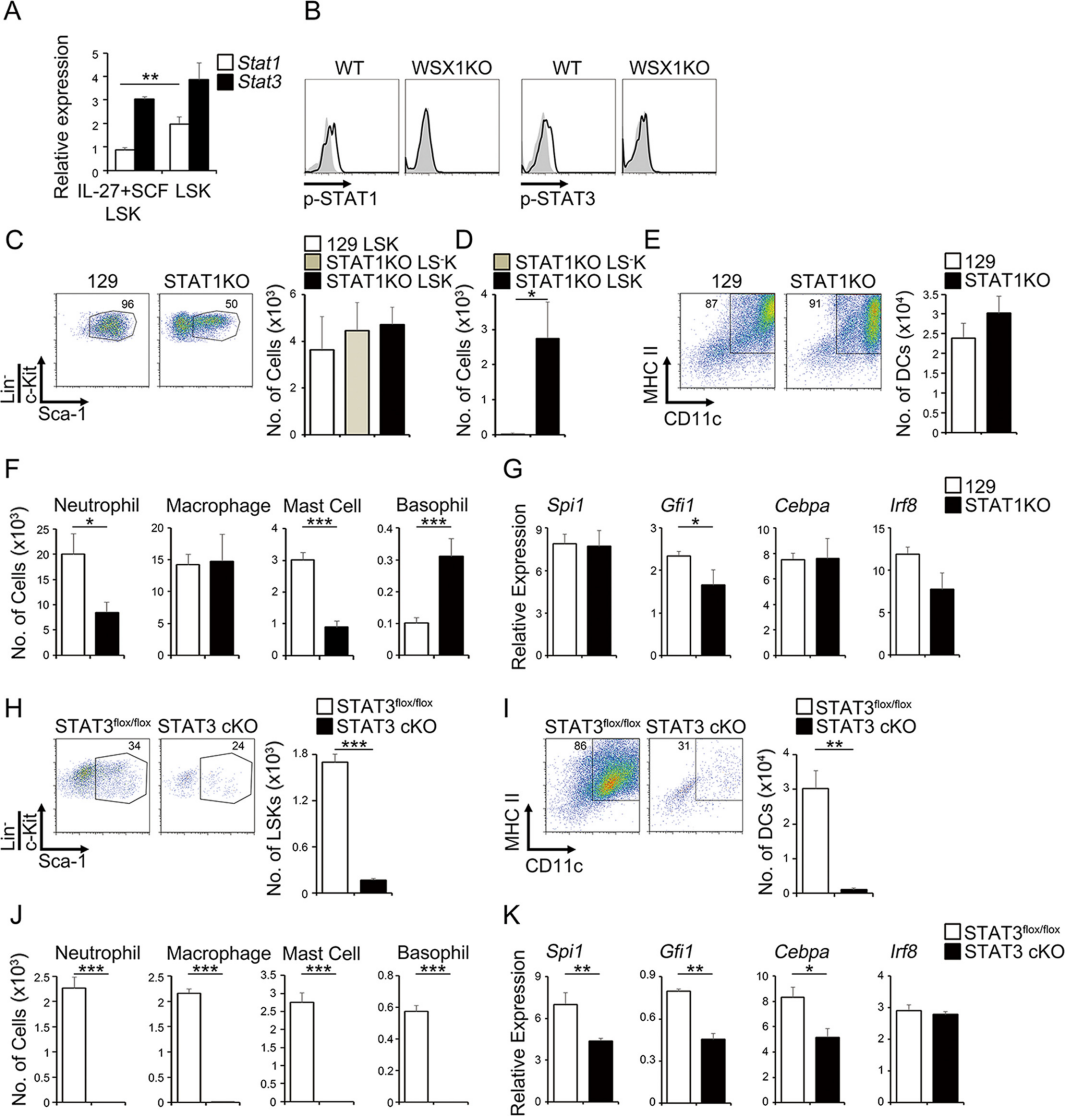
STAT1 and STAT3 are important for expansion and differentiation of LSK cells by IL-27 and SCF. (A) mRNA expression of *STAT1* and *STAT3* in the LSK cells expanded by IL-27 and SCF for 2 weeks and in primary LSK cells. (B) Flow cytometry histogram analysis of primary LSK cells after stimulation with IL-27 and SCF for 60 min using anti-pY-STAT1 or anti-pY-STAT3 (solid line) and control antibody (plain line with shading). (C-D) Dispensable role for STAT1 in the expansion of LSK cells in response to IL-27 and SCF. LSK cells (1 × 10^3^) purified from BM cells of WT (129) mice and *STAT1*-deficient mice were expanded by IL-27 and SCF for 2 weeks and analyzed for expression of LSK phenotype (C). The cell numbers of LSK and LS^−^K cell populations were counted. Purified cells of the *STAT1*-deficient LSK and LS^−^K cells (1 × 10^3^) were further stimulated by IL-27 and SCF for more 1 week and the cell number of expanded cells was counted (D). (E-G) Contribution of STAT1 to the differentiation into mDC (E) and myeloid cells (F) of LSK cells expanded by IL-27 and SCF for 2 weeks together with their mRNA expression of transcription factors (G). (H) Indispensable role for STAT3 in the expansion of LSK cells in response to IL-27 and SCF. Purified GFP^−^ *STAT3* ^*flox/flox*^ LSK cells and GFP^+^ *STAT3* cKO LSK cells (1 × 10^2^) were expanded by IL-27 and SCF for 10 days and analyzed for expression of LSK phenotype and their cell numbers. (I-K) Critical role for STAT3 in the differentiation into mDC (I) and myeloid cells (J) of LSK cells expanded by IL-27 and SCF for 10 days together with their mRNA expression of transcription factors (K). Data are shown as mean ± SEM (n = 3–4) and representative of two to three independent experiments. *P < 0.05, **P < 0.01, ***P < 0.005.

We and others previously demonstrated that IL-27 activates both STAT1 and STAT3 through WSX-1 and gp130, respectively [14,15]. Consistent with these reports, real-time RT-PCR analysis revealed that LSK populations purified from LSK cells expanded by IL-27 and SCF and primary BM cells were positive for mRNA expression of *STAT1* and *STAT3* (Fig 3A). Phosphorylation of STAT1 and STAT3 was also detected in primary WT LSK cells, but not *WSX-1*-deficient LSK cells (Fig 3B), in response to IL-27 and SCF, which were analyzed by flow cytometry. Furthermore, IL-27 alone induced phosphorylation of both STAT1 and STAT3, whereas SCF alone failed to induce phosphorylation of either one, as discussed previously [33] (S4 Fig).

To further investigate the roles of STAT1 and STAT3 in the expansion of LSK cells and the ability to differentiate into myeloid progenitors by IL-27 and SCF, we used *STAT1*-deficient LSK cells and conditional *STAT3*-knockout (*STAT3* cKO) LSK cells. LSK cells from WT (129) and *STAT1*-deficient mice were stimulated with IL-27 and SCF. Although WT LSK cells comprised more than 90% of cells with the LSK phenotype after 7 days, *STAT1*-deficient LSK cells comprised half LSK phenotype cells and half Sca-1^−^LK (LS^−^K) (Fig 3C). Nevertheless, the number of expanded cells was comparable among them, probably due to the anti-proliferative effects by STAT1 signaling [34]. However, the purified *STAT1*-deficient LS^−^K cells failed to survive thereafter, even in the presence of IL-27 and SCF (Fig 3D), although the purified *STAT1*-deficient LSK cells expanded well (Fig 3D), as did WT 129 LSK cells (Fig 3C). Moreover, WT and *STAT1*-deficient LSK cells had similar abilities to differentiate into MHC class II^+^CD11c^+^ mDC cells (Fig 3E) and macrophages (Fig 3F). *STAT1*-deficient LSK cells showed reduced ability to differentiate into neutrophils and mast cells, but increased ability to differentiate into basophils (Fig 3F). In line with the reduced ability to differentiate into neutrophils, mRNA expression of the critical transcription factor *Gfi1* was significantly reduced in *STAT1*-deficient LSK cells compared with that in WT LSK cells (Fig 3G). In contrast, *STAT3* cKO LSK cells expanded very little in response to IL-27 and SCF (Fig 3H). Moreover, the residual surviving LSK cells almost completely lost the ability to differentiate into mDCs (Fig 3I) and myeloid cells (Fig 3J). Consistent with the abrogated abilities, these cells showed reduced expression of the critical transcription factors such as *Spi1*, *Gfi1*, and *Cebpa*, but not *Irf8* (Fig 3K). These results suggest that both STAT1 and STAT3 are necessary for LSK cells to fully expand and differentiate into myeloid progenitor cells in response to IL-27 and SCF.

### IL-27 and SCF expand CD34^−^CD150^+^ LSK cells into myeloid progenitor cells

**Fig 4:**
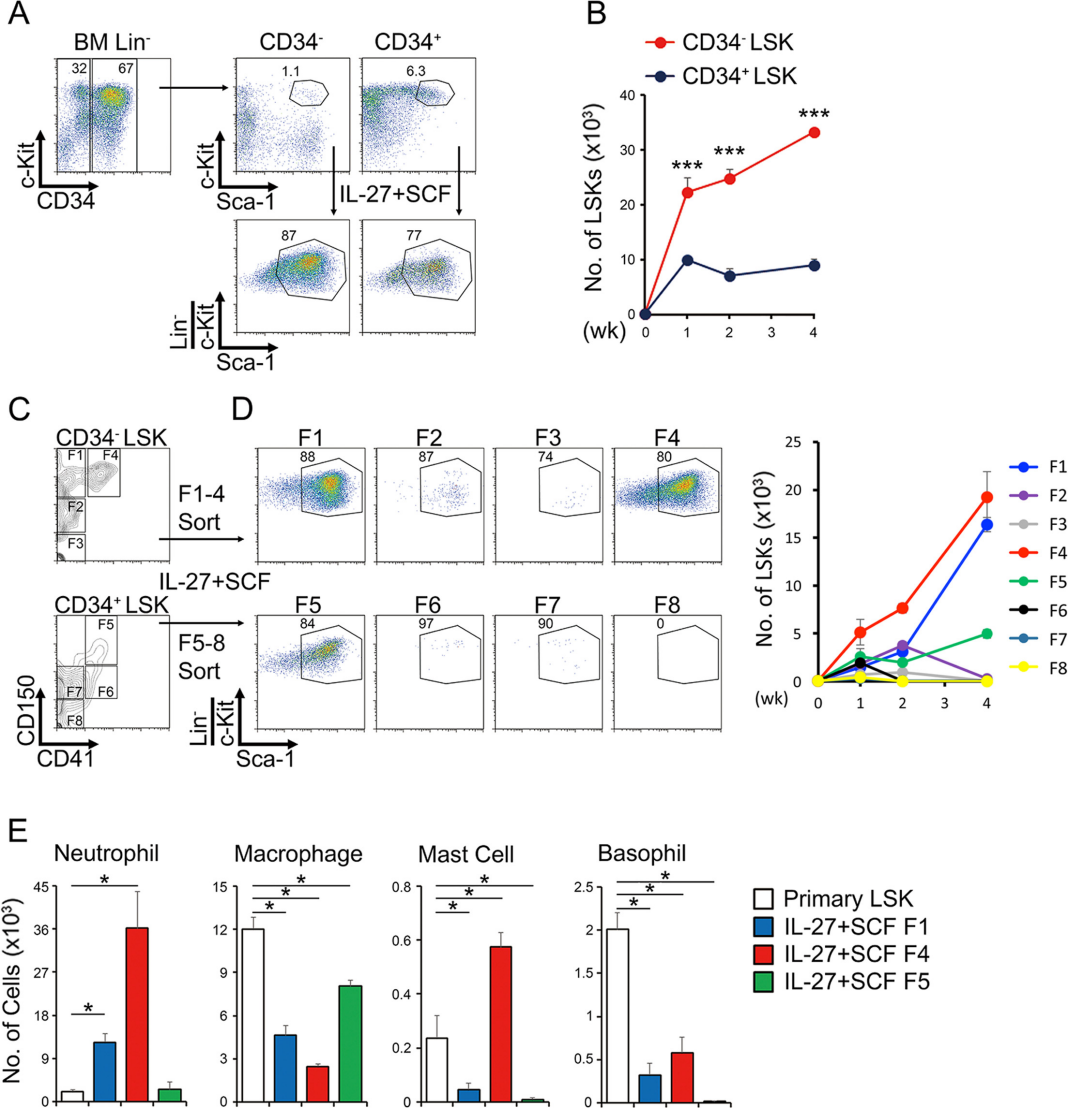
IL-27 and SCF expand CD34^−^CD150^+^ LSK cells into multipotent myeloid progenitor cells. (A-B) Enhanced expansion of CD34^−^ LSK cells by IL-27 and SCF. LSK cells from WT mice were divided into two populations according to the expression of CD34, CD34^−^ LSK, and CD34^+^ LSK cells, and each population (1 × 10^2^) was stimulated with IL-27 and SCF. One to 4 weeks later, these stimulated cells were analyzed by flow cytometry; representative dot plots of c-Kit and Sca-1 in the Lin^−^ population at 2 weeks are shown (A). Cell numbers of these stimulated cells were counted with time course (B). (C-E) Augmented expansion of CD34^−^CD150^+^ LSK cells by IL-27 and SCF. LSK cells were further divided into eight populations (F1-F8) according to the expression of CD34, CD150, and CD41 (C), and each population (50 cells) purified by sorting was stimulated with IL-27 and SCF. One to 4 weeks later, these stimulated cells were analyzed by flow cytometry. Representative dot plots of c-Kit and Sca-1 in the Lin^−^ population at 4 weeks are shown, and the cell number of the LSK cell population in these stimulated cells was counted with time course (D). LSK populations (1 × 10^3^) purified from primary or IL-27/SCF-expanded F1, F4, and F5 LSK cells were differentiated into myeloid cells by IL-3 and SCF, and cell number was counted (E). Data are shown as mean ± SEM (n = 3–4) and are representative of two to three independent experiments. *P < 0.05, ***P < 0.005.

To more precisely define which cell population responds to stimulation with IL-27 and SCF, LSK cells were further divided into two populations according to CD34 expression. Although the percentage of the more primitive population of CD34^−^ LSK cells was much less than that of CD34^+^ LSK cells, the CD34^−^ LSK cells responded much better to stimulation with IL-27 and SCF and expanded more vigorously than CD34^+^ LSK cells (Fig 4A and 4B). Then, the LSK cells were further divided into eight populations, F1 to F8, including LT-HSCs (CD34^−^CD150^+^CD41^−^ LSK, F1) and myeloid-restricted progenitor cells with long-term repopulating activity (MyRPs, CD34^−^CD150^+^CD41^+^ LSK, F4) according to the recently revised criteria [19]. Respective populations purified by sorting (Fig 4C) were stimulated with IL-27 and SCF. Only two populations, F1 and F4, vigorously expanded. F5, which corresponds to populations more differentiated toward myeloid cells such as macrophages, slightly expanded (Fig 4D and 4E). The F1 and F4 populations expanded by IL-27 and SCF had great abilities to differentiate into myeloid cells, particularly neutrophils (Fig 4E). Thus, IL-27 and SCF expand CD34^−^CD150^+^ LSK cells, including LT-HSCs and MyRPs, and differentiate them into myeloid progenitor cells, which have great potential to differentiate mainly into neutrophils.

### IL-27 plays an important role in expansion, differentiation, and mobilization of LSK cells to control malaria infection

**Fig 5:**
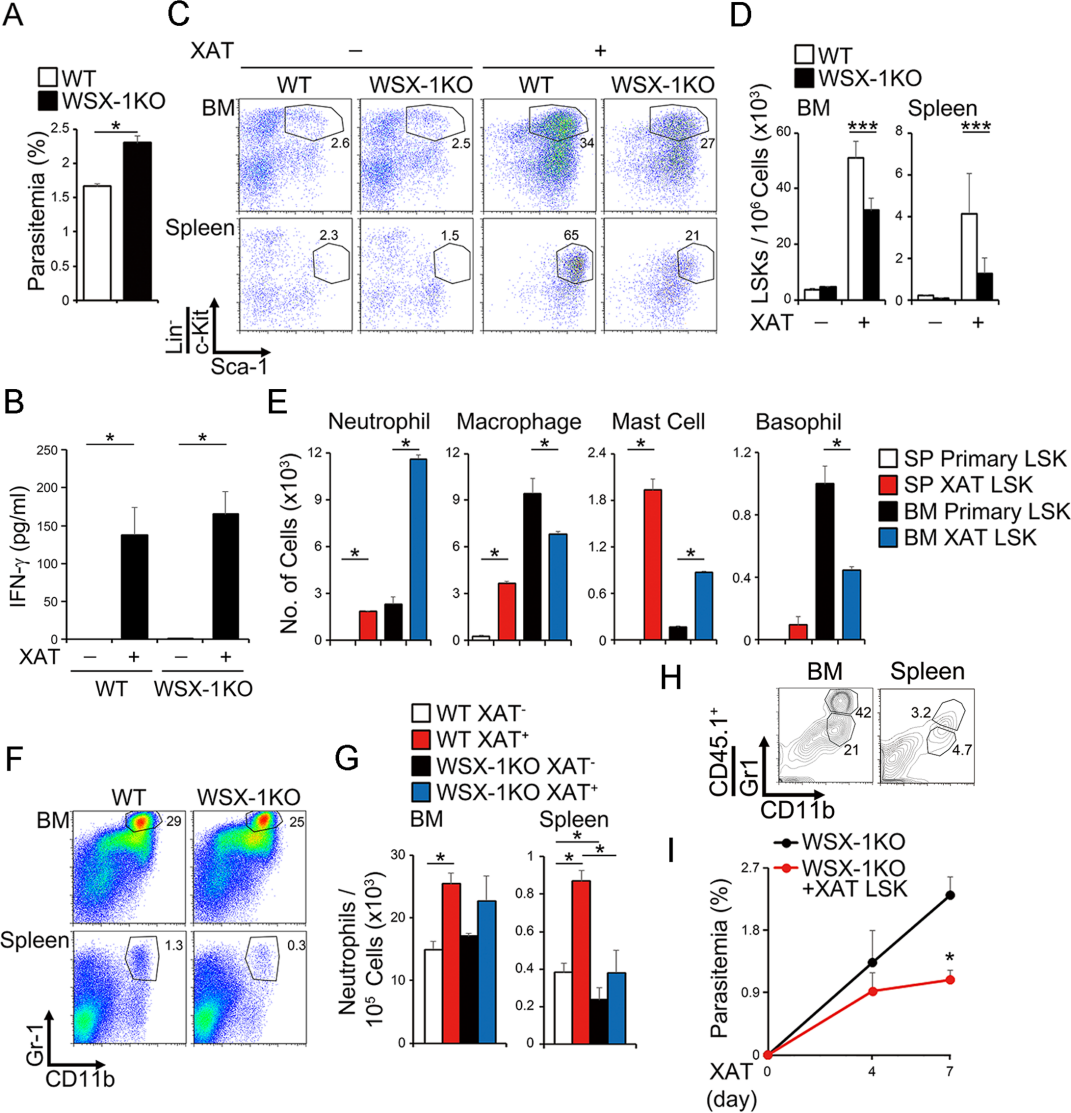
IL-27 plays an important role in expansion, differentiation, and mobilization of LSK cells to control malaria infection. (A-D) Reduced induction of LSK cell population in *WSX-1*-deficient mice after malaria infection, accompanied by increased parasitemia and comparable production of IFN-γ. WT or *WSX-1*-deficient mice were infected with the blood stage of *P*. *berghei* XAT. Seven days later, parasitemia (A) and serum IFN-γ level (B) were determined, and LSK populations in the BM and spleen were analyzed by flow cytometry, and representative dot plots of c-Kit and Sca-1 in the Lin^−^ population are shown (C). Cell number of the LSK cell population was counted (D). (E) Augmented potential of LSK cells to differentiate into myeloid cells by malaria infection. LSK cells (1 × 10^3^) in the BM and spleen of the malaria-infected or non-infected WT mice were purified and differentiated into myeloid cells *in vitro* by IL-3 and SCF, and cell number of differentiated cells was counted. (F-G) Reduced cell number of neutrophils in *WSX-1*-deficient mice after malaria infection. The BM and spleen cells were analyzed for expression of Gr-1 and CD11b at 7 days after the infection (F), and cell number of neutrophils (Gr-1^+^CD11b^+^) was counted (G). (H-I) Decreased parasitemia in the *WSX-1*-deficient mice transferred with LSK cells purified from BM cells of the malaria-infected WT mice. LSK cells purified from BM cells of the infected WT CD45.1 mice were transferred into non-lethally irradiated *WSX-1*-deficient CD45.2 mice 7 days before infection. Neutrophil population in the BM and spleen was analyzed by flow cytometry, and representative dot plots of CD11b and Gr-1 in the CD45.1^+^ population are shown (H). Parasitemia was measured 4 and 7 days after the infection (I). Data are shown as mean ± SEM (n = 3–9) and are representative of at least two independent experiments. *P < 0.05, ***P < 0.005.

We previously demonstrated that in the blood stage of malaria infection with the attenuated variant *Plasmodium (P) berghei* XAT derived from the lethal strain *P*. *berghei* NK65 IFN-γ production induced by IL-12 and phagocytic cells in the spleen are critical for controlling parasitemia [35,36]. Recently, it was reported that the blood stage of *P*. *chabaudi* infection induces mobilization of early myeloid progenitor cells out of BM, thereby transiently establishing myelopoiesis in the spleen through IFN-γ to resolve the infection [6,37]. In line with these results, *WSX-1*-deficient mice showed more increased parasitemia than WT mice at 7 days (Fig 5A), just prior to when the parasitemia reaches its peak after infection with *P*. *berghei* XAT (S5A Fig). In contrast, no significant difference was observed in the serum IFN-γ levels in WT and *WSX-1*-deficient mice (Fig 5B). The infection markedly induced the enhanced percentage and number of LSK cells in the BM and spleen of WT mice (Fig 5C and 5D). The LSK cells in the BM showed greatly augmented abilities to differentiate *in vitro* into neutrophils and mast cells, but had slightly reduced abilities to differentiate into macrophages and basophils after infection (Fig 5E). Moreover, the LSK cells in the spleen exhibited a much more enhanced ability to differentiate into neutrophils, macrophages, and mast cells (Fig 5E). In contrast, of note, *WSX-1*-deficient mice showed significantly reduced percentage and number of LSK cells in the BM and spleen compared with WT mice after infection (Fig 5C and 5D). In particular, the cell number of the neutrophils in the spleen was increased very little in *WSX-1*-deficient mice (Fig 5F and 5G). Consistent with this, LSK cells from BM of *WSX-1*-deficient mice showed reduced abilities to differentiate *in vitro* into neutrophils after infection compared with those of WT mice (S6 Fig). In addition, more greatly reduced abilities to differentiate *in vitro* into various myeloid cells were observed when LSK cells from spleen were used (S6 Fig). Moreover, mixed bone marrow chimera experiments using bone marrow cells from WT and *WSX-1*-deficient mice revealed that the effect of IL-27 on the expansion of LSK cells and neutrophils is actually a cell-autonomous direct effect (S7 Fig).

To further elucidate the protective role of LSK cells in malaria infection, LSK cells purified from BM cells of infected WT CD45.1 mice were injected into the infected *WSX-1*-deficient CD45.2 mice. Consistent with the increased percentage of neutrophils differentiated from the WT LSK cells in the BM and spleen of transferred *WSX-1*-deficient mice (Fig 5H), parasitemia was significantly decreased in the *WSX-1*-deficient mice by the transfer of WT LSK cells compared with that in non-transferred *WSX-1*-deficient mice (Fig 5I). Thus, the blood stage of malaria infection induces expansion, differentiation, and mobilization of LSK cells into the spleen to produce myeloid cells such as neutrophils in an IL-27-dependent manner.

### Malaria infection enhances IL-27 expression through IFN-γ production to promote the expansion, differentiation, and mobilization of LSK cells

**Fig 6:**
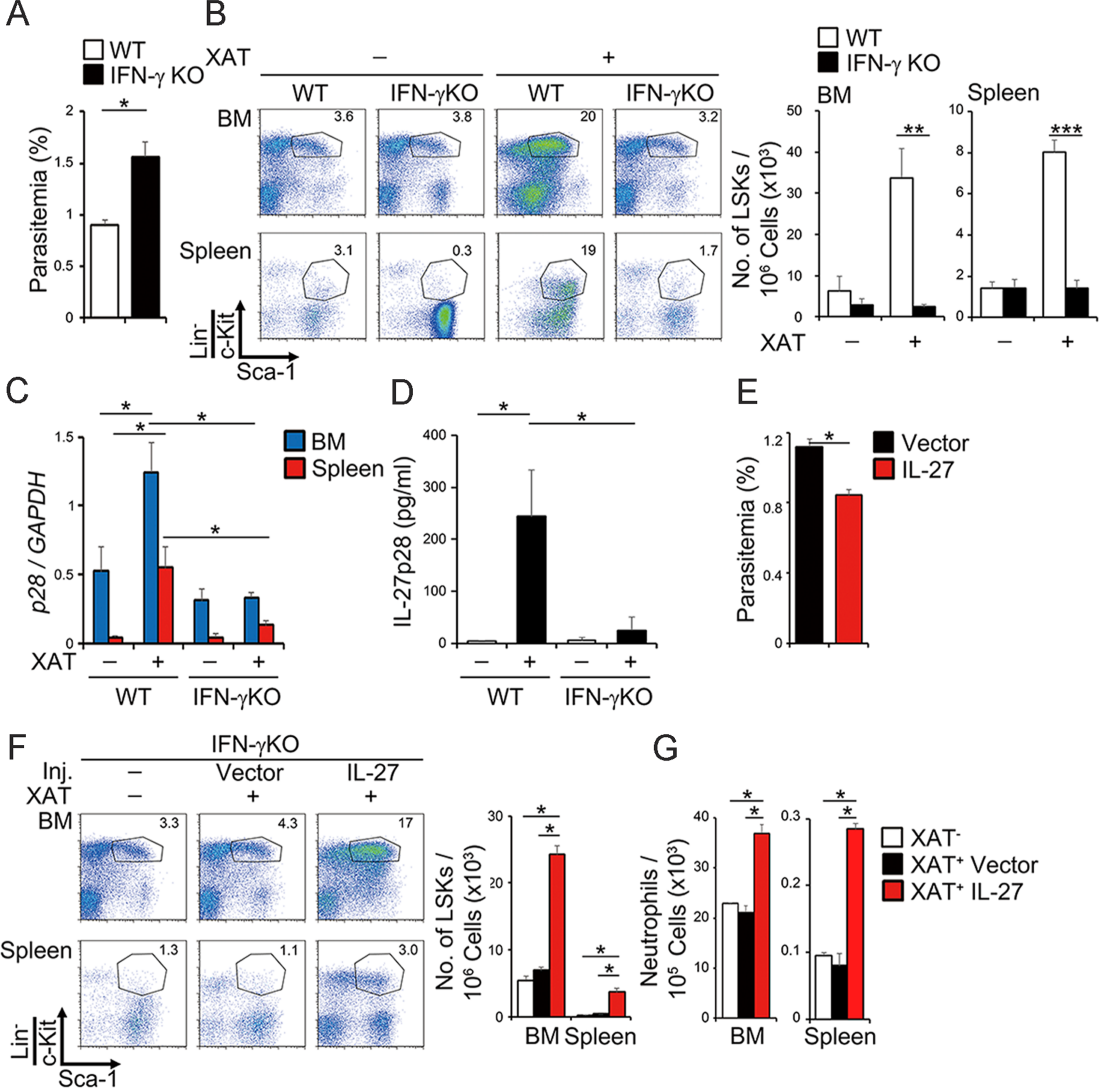
Malaria infection enhances IL-27 expression through IFN-γ production to promote the expansion, differentiation, and mobilization of LSK cells. (A-B) Indispensable role for IFN-γ in the expansion of LSK cells after malaria infection. WT and *IFN-γ*-deficient mice were infected with the blood stage of *P*. *berghei* XAT. Seven days later, parasitemia was determined (A) and LSK populations were analyzed by flow cytometry (B). (C-D) IFN-γ-dependent induction of IL-27 p28 subunit expression by malaria infection. RNA was prepared 7 days after the infection and analyzed for expression of *p28* by real-time RT-PCR (C), and serum p28 levels were determined by ELISA (D). (E-G) Decreased parasitemia and augmented expansion of LSK cell population in *IFN-γ*-deficient mice by IL-27. *IFN-γ*-deficient mice were hydrodynamically injected with IL-27-expression vector or control vector at days 0 and 4 after infection; at day 7, parasitemia was measured (E), LSK population was analyzed by flow cytometry (F), and cell numbers of LSK cells and neutrophils were counted (G). Data are shown as mean ± SEM (n = 3–5) and are representative of at least two independent experiments. *P < 0.05, **P < 0.01, ***P < 0.005.

Although it was previously reported that IFN-α and IFN-γ induce proliferation of HSCs *in vivo* [3–5], IFN-α and IFN-γ augmented the expansion of LSK cells very little *in vitro*, and only IL-27 enhanced it vigorously over 4 weeks (Fig 1G and 1H). However, similar to the work previously reported [37,38], *IFN-γ-*deficient mice showed increased parasitemia with almost no increase in the number of LSK cells in BM and spleen (Fig 6A and 6B). To clarify the molecular mechanism whereby IFN-γ induces the expansion of LSK cells, the expression of IL-27 subunits EBI3 and p28 was examined. Although the infection did not increase *EBI3* mRNA expression in the BM and spleen of both WT and *IFN-γ*-deficient mice (S8 Fig), intriguingly, the infection greatly enhanced *p28* mRNA expression in WT mice but failed to enhance it in *IFN-γ-*deficient mice (Fig 6C). In agreement with this, p28 protein levels in the serum were greatly increased by the infection in WT mice but not in *IFN-γ*-deficient mice (Fig 6D). Consistent with the *in vivo* role of IFN-γ, we also observed the augmentation of mRNA expression of *p28*, but not *EBI3*, and p28 protein production in the culture supernatants of WT BM cells stimulated with IFN-γ *in vitro* (S9 Fig).

To further clarify the role of IL-27 downstream of IFN-γ, we next performed the experiment to see the effects of forced expression of IL-27 on the susceptibility to malaria infection in *IFN-γ*-deficient mice. The hydrodynamic injection of IL-27 expression vector into the infected *IFN-γ*-deficient mice showed significantly decreased parasitemia compared with that of control vector (Fig 6E). This phenomenon was accompanied by the enhanced percentage of LSK cells and augmented numbers of LSK cells and neutrophils in both BM and spleen (Fig 6F and 6G). Thus, the blood stage of malaria infection augments the expression of IL-27 through IFN-γ, and IL-27 then promotes the expansion, differentiation, and mobilization of LSK cells into the spleen to control parasitemia.

## Discussion

Previously, we found that IL-27, which is in the IL-6/IL-12 family of cytokines, plays a role in the regulation of HSCs *in vitro* and *in vivo* [17]. Here, we have further elucidated that IL-27 is a unique cytokine that directly acts on LSK cells to promote their differentiation into myeloid progenitor cells called M-CSFR^+^Flt3^−^CD16/32^+^ LSK cells, which still retain the LSK phenotype (Fig 2A–2I). These progenitors have great potential to give rise to neutrophils, mDCs, and mast cells, but not to pDCs, cDCs, T cells, and B cells. Interestingly, among various BM progenitor cells, IL-27 and SCF vigorously and continuously expand only HSCs and primitive myeloid progenitor cells with long-term repopulating activity, such as LT-HSCs and MyRPs, respectively [19], for more than 4 weeks (Fig 4). Consistent with the ability to differentiate into myeloid progenitor cells, the LSK cells expanded by IL-27 and SCF expressed transcription factors such as *Spi1*, *Gfi1*, *Cebpa*, and *Cebpb*, which are critical for myeloid differentiation [25–27,30–32] (Fig 2J and S3 Fig). Although *Cebpb* was reported to be an important transcription factor for emergency granulopoiesis [30–32], STAT3 signaling was revealed to be important for its upregulation, whereas STAT1 signaling unexpectedly suppressed its expression (S10 Fig). This phenomenon seems to correlate to the expression level of the anti-apoptotic gene *Bcl-2* [39,40], but not the transcription factor *E2-2*, which is critical for pDC differentiation [41]. Further studies are necessary to elucidate the precise roles of each STAT in the regulation of *Cebpb* expression. Thus, IL-27 is one of the limited unique cytokines that directly acts on the most primitive LT-HSCs; it promotes their expansion and differentiation into myeloid progenitor cells, presumably through MyRPs [19], to replenish myeloid cells such as neutrophils in the periphery during emergency myelopoiesis.

Sca-1 is an IFN-responsive molecule that is highly upregulated in many hematopoietic cells following exposure to IFNs [10,11,37]. Consequently, myeloid-restricted progenitor cells normally identified as Lin^−^Sca-1^−^c-Kit^+^ (LS^−^K) become positive for Sca-1 and can no longer be distinguished from the real multipotent LSK cells, resulting in overestimation of the latter population, and this is a problem. IL-27 was previously reported to enhance the expression of Sca-1 on T cells [42]. However, to alleviate the problem, we initially identified the cell population responsive to IL-27 and SCF among BM cells by using LSK cells and various hematopoietic progenitor cells purified by sorting. Intriguingly, it turned out that the SCF and LSK cell populations expanded vigorously and continuously in response to IL-27, and that the LS^−^K cell populations including GMP, CMP, and MEP only transiently and slightly responded during the first week and then disappeared thereafter (Fig 1A–1E). Moreover, in almost all *in vitro* experiments, we used primary LSK cells that were purified by sorting. The high responsiveness of the LSK cells to IL-27 seems to be partially due to the higher mRNA expression of *WSX-1* in the LSK cells compared to that of other hematopoietic progenitor cells (S3 Fig) [17].

We previously demonstrated that during the blood stage of malaria infection with attenuated *P*. *berghei* XAT, IL-12-mediated IFN-γ production and phagocytic cells (including neutrophils) in the spleen are critical for controlling parasitemia [35,36,43]. Previous studies demonstrated that neutrophils play an important role in killing malaria parasites in mice, rats, and humans [43–45]. In marked contrast, regarding infection with lethal *P*. *berghei* NK65, IL-12-mediated IFN-γ production was shown to contribute to T-cell-dependent immunopathology [46]. However, a major role of IL-27 in infection is its suppression of excess immune responses against infection by controlling the production of pro-inflammatory cytokines [14–16]. Consistent with this, WSX-1/IL-27 was recently demonstrated to have a critical role in limiting the effector CD4^+^ T-cell-mediated immunopathology caused by IL-12-dependent IFN-γ production during infection with lethal *P*. *berghei* NK65 [47–49]. The present study clearly revealed that WSX-1/IL-27 contributes to clearance of parasites due to enhanced myelopoiesis during the early phase of infection with attenuated *P*. *berghei* XAT (Fig 5 and S5A Fig). However, during the late phase of infection, WSX-1/IL-27 seems to play a role in limiting the production of pro-inflammatory cytokines such as IFN-γ (S5B Fig), leading to augmented reduction of parasitemia (S5A Fig), as in the case of infection with lethal *P*. *berghei* NK65 [47–49]. Moreover, our preliminary data revealed that there were no apparent differences observed in parasitemia or expansion of LSK cells and neutrophils in the BM and spleen of WT and *WSX-1*-deficient mice 7 days after infection with lethal *P*. *berghei* NK65 (S11 Fig). It is conceivable that pro-inflammatory cytokines other than IL-27 were abundantly produced in the absence of IL-27 during infection with lethal *P*. *berghei* NK65 and that late-phase infection with attenuated *P*. *berghei* XAT may have redundantly compensated for the loss of IL-27 to promote myelopoiesis. However, other studies have shown that IL-27 limits migration of neutrophils from the BM to the site of inflammation by reducing production of cytokines and chemokines during influenza infection [50] and septic peritonitis [51]. IL-27 was also reported to be a negative regulator of neutrophil function [52]. Although IL-27 directly promotes myelopoiesis to produce myeloid progenitors in BM, as shown in the present study, IL-27 may indirectly regulate migration of these progenitors and neutrophils to the site of inflammation and limit neutrophil function. Thus, IL-27 has both positive and negative effects on neutrophils; therefore, the overall outcome of the effects of IL-27 is likely to be governed by the balance between these effects, depending on the disease model.

It was recently demonstrated that *P*. *chabaudi* infection induces mobilization of early myeloid progenitor cells out of BM, thereby transiently establishing myelopoiesis in the spleen through IFN-γ [37]. However, the expression of IFN-γR in the hematopoietic compartment was dispensable, whereas its expression in the irradiation-insensitive cellular compartment, including endothelial cells and stromal cells, was important [37]. Secretion of IFN-γ-induced chemokines such as CCL2 and CCL7 by non-hematopoietic cells plays a critical role in the mobilization of CCR2-expressing HSCs [37]. In this study, however, there is no experimental evidence regarding how IFN-γ regulates the activation of HSCs. In the present study, *WSX-1*-deficient mice showed significantly reduced numbers of LSK cells and neutrophils compared with WT mice after *P*. *berghei* XAT infection, resulting in increased parasitemia (Fig 5A–5F and S5A Fig). These results suggest that endogenous IL-27 greatly contributes to the clearance of parasitemia through augmentation of myelopoiesis. Moreover, similar to *P*. *chabaudi* infection, *P*. *berghei* XAT infection could not increase the number of LSK cells in *IFN-γ*-deficient mice with increased parasitemia (Fig 6A and 6B). Of note, after *P*. *berghei* XAT infection, *p28* mRNA expression and its serum protein level were markedly upregulated in an IFN-γ-dependent manner (Fig 6C and 6D and S9 Fig). This is consistent with previous reports indicating that *p28* gene transcription in macrophages is induced by IFN-γ and TLR ligands [53], and that IFN-γ limits Th17-mediated and Th9-mediated autoimmune inflammation through IL-27 production [54,55]. In addition, the hydrodynamic injection of the IL-27 expression vector into infected *IFN-γ*-deficient mice greatly recovered the number of LSK cells and neutrophils in the BM and spleen and eventually reduced parasitemia (Fig 6E–6G). Thus, during malaria infection, it is highly conceivable that the proliferative effects on LSK cells by IFN-γ are indirectly mediated by IL-27. In our study, we could not observe any direct proliferative effect of IFN-α and IFN-γ on LSK cells *in vitro*, as recently pointed out by others [10,11], and only IL-27 augmented the proliferative effect for more than 4 weeks (Fig 1G and 1H).

Recently, IL-27 was reported to have a polyglutamic acid domain in the p28 subunit, which is unique among cytokines, and to confer hydroxyapatite-binding and bone-binding properties and bone tropism to bone sialoprotein and the endosteal bone surface [56]. This location in the BM has been identified as a niche for HSCs [57], and these properties support the idea that IL-27 plays a critical role in the regulation of HSCs in that niche. We detected much higher expressions of IL-27 subunits (both *p28* and *EBI3*) at mRNA levels in BM than in the spleen during the steady state, and *P*. *berghei* XAT infection greatly augmented the expression of *p28* mRNA in both BM and spleen, and also its serum protein level (Fig 6C and 6D). Further studies are necessary to clarify which BM cells produce IL-27 during malaria infection; mesenchymal stromal cells might be a candidate because of their reported IL-6 production during viral infection [12], as described in the next section.

It was recently demonstrated that specific cytotoxic CD8^+^ T cells during an acute viral infection with lymphocytic choriomeningitis virus secrete IFN-γ, thus enhancing the production of IL-6 in BM mesenchymal stromal cells and resulting in an increased number of early multipotent progenitors and committed myeloid precursors in the BM and accumulation of myeloid cells in the periphery [12]. The IL-6Rα chain is only expressed at the stage of early multipotent progenitors and downstream myeloid precursors, and it is lacking HSCs [12]. In contrast, IL-27 most predominantly acts on only HSCs, as shown in the present study. Both IL-6 and IL-27 share gp130, which is ubiquitously expressed as a common receptor subunit. Therefore, downstream of IFN-γ, both IL-27 and IL-6 may be necessary to induce the maximum myelopoiesis to control infection. However, the mode of IL-6 action is complex and there are two major mechanisms: IL-6 classic signaling through membrane IL-6Rα and IL-6 *trans*-signaling through soluble IL-6Rα [58,59]. It was recently reported that *IL-6Rα*-deficient mice show increased resistance to *P*. *chabaudi* infection and that IL-6 *trans*-signaling, but not IL-6 classic signaling, contributes to a lethal outcome of infection [60]. In contrast to the viral infection, we could not detect any increased mRNA expression of *IL-6* in the BM or spleen of WT and *IFN-γ*-deficient mice with *P*. *berghei* XAT infection (S8 Fig). A similar inability of IFN-γ to enhance *IL-6* mRNA expression in WT BM cells *in vitro* was also observed (S9A Fig). In addition, *IL-6*-deficient mice showed little increased susceptibility to the *P*. *berghei* XAT infection, reduced cell numbers in the LSK cell population, and reduced neutrophils in the BM and spleen (S12 Fig). Thus, individual pathogens may utilize different mechanisms to induce emergency myelopoiesis through IL-27, IL-6, and others.

In conclusion, the present results provide a novel role and mechanism for the action of IL-27 downstream of IFN-γ in the efficient expansion of myeloid progenitor cells from LT-HSCs and MyRP cells and their mobilization into the spleen during acute malaria infection.

## Materials and Methods

### Ethics statement

The animal study was approved by the Animal Care and Use Committee of Tokyo Medical University (S-230043, S-24012, S-25059, S-26003, and S-27009) and was performed in accordance with our institutional guidelines and the Fundamental Guidelines for Proper Conduct of Animal Experiment and Related Activities in Academic Research Institutions under the jurisdiction of the Ministry of Education, Culture, Sports, Science and Technology, 2006.

### Mice

C57BL/6 (CD45.2) mice and C57BL/6 (CD45.1) mice were purchased from Sankyo Lab (Tokyo, Japan). The 129/Sv mice and *STAT1*-deficient mice (129/Sv background) were purchased from Taconic Farms (Germantown, NY, USA). *IL-6*-deficient mice (C57BL/6 background) were purchased from Jackson Laboratory (Bar Harbor, ME, USA). *IFN-γ*-deficient mice (C57BL/6 background), *STAT3* ^*flox/flox*^ mice (a mixed background of 129/Sv and C57BL/6), and *GFP* Tg mice (C57BL/6 background) [61] were provided by Dr. Iwakura (Tokyo University of Science), Dr. Takeda (Osaka University), Dr. Okabe (Osaka University) and Dr. Ito (Tokyo Medical University), respectively. In addition to these mice, *IL-27* Tg mice (C57BL/6 background) [17], and *WSX-1*-deficient mice (C57BL/6 background) [62] were maintained in specific pathogen-free conditions under the care of the Laboratory Animal Center of Tokyo Medical University. *STAT3* cKO cells were obtained by infecting *STAT3* ^*flox/flox*^ cells with Cre-expressing retrovirus *in vitro*.

### Antibodies and reagents

Monoclonal antibodies (mAbs) for mouse c-Kit (2B8), Sca-1 (D7), CD3ε (145-2C11), CD4 (GK1.5), CD8α (53–6.7), CD19 (6D5), CD49b (DX5), Gr-1 (RB6-8C5), TER119/erythroid cell (TER-119), CD11c (N418), CD11b (M1/70), F4/80 (BM8), NK1.1 (PK136), B220 (RA3-6B2), FcεRIα (MAR-1), M-CSFR (AFS98), Flt3 (A2F10), CD16/32 (2.4G2), CD150 (TC15-12F12.2), CD41 (MWReg30), IL-7Rα (A7R34), MHC class II I-A/I-E (M5/114.15.2), Siglec H (551), Ly6G (1A8), CD45.1 (A20), and CD45.2 (104) were purchased from BioLegend (San Diego, CA). mAbs against mouse pY701-STAT1 (4a) and pY705-STAT3 (4/P-STAT3) were purchased from BD Pharmingen (San Diego, CA). mAbs against mouse CD34 (RAM34) was purchased from eBioscience (La Jolla, CA). mAbs against mouse PDCA1 (JF05-1C2.4.1) was purchased from Miltenyi Biotec (Bergisch Gladbach, Germany). APC-Cy7-conjugated streptavidin, PerCP/Cy5.5-conjugated streptavidin, and Brilliant Violet 510-conjugated streptavidin were purchased from BioLegend and used to reveal staining with biotinylated Abs. Mouse recombinant IL-27 and hyper-IL-6 were prepared as a tagged single-chain fusion protein by flexibly linking EBI3 to p28 and soluble IL-6Rα to IL-6, respectively, using HEK293-F cells (Life Technologies, Carlsbad, CA) as described previously [63,64]. Mouse recombinant SCF, IL-1β, IL-3, IL-6, IL-7, IL-11, IL-12, thymic stromal lymphopoietin (TSLP), G-CSF, M-CSF, GM-CSF, TNF-α, and human recombinant TPO were purchased from PeproTech (Rocky Hill, NJ). Human recombinant Flt3L was purchased from Miltenyi Biotec. Mouse recombinant IL-23, IL-25, and IL-33 were purchased from R&D Systems (Minneapolis, MN). Mouse IFN-α was purchased from PBL Biomedical Laboratories (Piscataway, NJ). Mouse recombinant IFN-γ was provided from Shionogi Pharmaceutical Co., Ltd. (Osaka, Japan),

### Preparation of cells

Spleen and BM Lin^−^ cells were enriched by negative selection using an autoMACS Pro (Miltenyi Biotec) with a combination of magnetic beads conjugated with mAbs against CD3ε, CD4, CD8α, Gr-1, TER119, CD11b, CD11c, NK1.1, B220, and FcεRIα. Subsequently, cells were stained with mAbs against c-Kit, and Sca-1 was used for LSK. For multiple fractions of HSC, cells were stained with CD34, c-Kit, Sca-1, CD150, and CD41 mAbs [19]. In the case of common lymphoid progenitor (CLP), macrophage-DC progenitor (MDP), and common DC progenitor (CDP), cells were stained with c-Kit, Sca-1, IL-7Rα, M-CSFR, and Flt3 mAbs [20–22]. For GMP, CMP, and MEP, cells were stained with CD34, c-Kit, Sca-1, and CD16/32 mAbs [20–22]. Sorting was performed on FACS Aria or FACS Aria III (BD Bioscience).

### Culture of cells

Cells were cultured at 37°C under 5% CO_2_/95% air in RPMI-1640 (SIGMA, St. Louis, MO) containing 10% fetal calf serum, 50 μM 2-mercaptoethanol (GIBCO, Grand Island, NY), and 100 μg/ml kanamycin (Meiji Seika, Tokyo, Japan). To proliferate progenitors, sorted cells were cultured with 10 ng/ml IL-27 and/or 10 ng/ml SCF. To examine the effect of various cytokines on proliferation of LSK, IL-1β, IL-3, IL-6, hyper-IL-6, IL-11, IL-12, IL-23, IL-25, IL-33, TSLP, G-CSF, TPO, TNF-α, and IFN-α were used as a final concentration of 10 ng/ml. IFN-γ was used as a final concentration of 100 U/ml. Half of the medium was changed every 3 days, with cytokines added.

### *In vitro* differentiation assay

For evaluation of mDC potential, sorted cells (1–5 × 10^3^) in 96-well plates were cultured with 20 ng/ml GM-CSF for 3 to 10 days. For pDC and cDC potential, sorted cells (5 × 10^3^) were cultured with 100 ng/ml Flt3L and 20 ng/ml TPO for 10 days. For analysis of multipotent myeloid potential, sorted cells (1 × 10^2^–5 × 10^3^) in 96-well plates were cultured with 10 ng/ml SCF and 10 ng/ml IL-3 for 6 days. Half of the medium was replaced every 3 days, with cytokines added. For detection of B-cell potential, sorted cells (2 × 10^2^) were cultured with a monolayer of thymic stromal cells (TSt4) containing 2 ng/ml IL-7 for 15 days [24]. For detection of T-cell potential, sorted cells (2 × 10^2^) were cultured with TSt4 cells expressing DLL1, TSt4/DLL1 cells, containing 2 ng/ml IL-7 and 2 ng/ml Flt3L for 18 days [24]. Half of the medium was replaced every 7 days, with cytokines added. The following cell surface markers were used to identify respective cells: mDC; MHC class II^+^CD11c^+^, pDC; Siglec H^+^PDCA1^+^CD11c^+^, cDC; Siglec H^−^PDCA1^−^CD11c^+^, neutrophil; Ly6G^+^CD11b^+^ or Gr-1^+^CD11b^+^, macrophage; F4/80^+^CD11b^+^, mast cell; c-Kit^+^FcεR1α^+^CD11b^−^, basophil; CD49b^+^FcεR1α^+^CD11b^−^, B cell; B220^+^CD19^+^, double positive T cell; and CD4^+^CD8^+^.

### Flow cytometry

Flow cytometry was performed on a FACS Canto II (BD Bioscience, San Jose, CA) and data were analyzed using FlowJo Software (Tree Star, Ashland, OR). Cell number was counted using flow cytometry unless otherwise indicated. For intracellular cytokine staining, cells were fixed with Fixation Buffer (BD Bioscience) for 30 min and permeabilized with Perm Buffer II (BD Bioscience) for 30 min. Then, samples were stained with antibodies for pY701-STAT1 and pY705-STAT3.

### Quantitative real-time RT-PCR

Total RNA was prepared using RNeasy Mini Kit (QIAGEN, Hilden, Germany), and cDNA was prepared using oligo(dT) primer and SuperScript III RT (Invitrogen, Carlsbad, CA, USA). Real-time quantitative PCR was performed using SYBR Premix Ex Taq II and a Thermal cycler Dice real-time system according to the manufacturer’s instructions (TAKARA, Otsu, Shiga, Japan). *Glyceraldehyde-3-phosphate* (*GAPDH*) was used as housekeeping gene to normalize mRNA. Relative expression of real-time PCR products was determined by using the ΔΔCt method to compare target gene and *GAPDH* mRNA expression. Primers used in this study are listed in S1 Table.

### Adoptive cell transfer

For *in vivo* proliferation analysis, BM LSK cells (8 × 10^3^) purified from *GFP* Tg mice were intravenously transferred into irradiated (4 Gy) WT and *IL-27* Tg mice. To evaluate *in vivo* development, IL-27/SCF-cultured BM LSK cells (2 × 10^4^) from CD45.1 congenic mice were transplanted into sublethally irradiated (6 Gy) CD45.2 recipient mice with the same congenic BM cells (2 × 10^6^). As in the case of malaria infection, BM LSK cells (5 × 10^4^) were sorted from malaria-infected CD45.1 mice and intravenously transferred into irradiated (4 Gy) *WSX1*-deficient mice 7 days before infection.

### Retroviral transduction

The retroviral vector pMX-Cre-GFP (from Dr. M. Kubo) was transfected into Platinum-E packaging cells [65] using FuGENE 6 (Promega, Madison, WI), and supernatants of these cultures were used as the source of viral particles. LSK cells sorted from BM cells of *STAT3* ^*flox/flox*^ mice were stimulated with IL-27 and SCF (10 ng/ml each) and transduced with viral particles by spin infection (2,000 rpm, 90 min, 25°C) using 8 μg/ml Polybrene at 24 hr and 48 hr later. The next day, GFP^+^ LSK cells were sorted and used as *STAT3* cKO LSK cells.

### Malaria infection

Mice were injected intravenously with a red blood cell (RBC) suspension containing parasitized RBC (1 × 10^4^) with the nonlethal strain *P*. *berghei* XAT [35], which is an irradiation-induced attenuated variant of the lethal strain *P*. *berghei* NK65, or the *P*. *berghei* NK65 [46]. Parasitemia was assessed by the microscopic examination of Giemsa-stained smears of tail blood after infection. The percentage of parasitemia was calculated as follows: parasitemia (%) = [(number of infected RBC) / (total number of RBC counted)] × 100.

### Hydrodynamic tail-vein injection

*IFN-γ*-deficient mice were intravenously injected with 25 μg of p3xFLAG-CMV (Sigma Chemical Co., St. Louis, MO), or p3xFLAG-IL-27 plasmids at days 0 and 4 after malaria infection.

### ELISA

Amounts of IL-27 p28 in culture supernatants or serum were determined by using Quantikine kits (R&D) according to the manufacturer’s instruction.

### Statistical analysis

Data are represented as mean ± SEM. Statistical analyses were performed by two-tailed Student’s *t* test for two groups, and by one-way ANOVA and Bonferroni’s multiple comparison tests for multiple groups. *P* < 0.05 was considered to indicate a statistically significant difference.

## Supporting Information

S1 Table Primers used in this study. (TIF)

S1 Fig IL-27 possesses the strongest ability to augment the expansion of LSK cells retaining the LSK phenotype among various cytokines. LSK cells (5 × 10^2^) from WT mice were stimulated by various cytokines together with SCF. The stimulated and expanded cells were analyzed for expression of c-Kit and Sca-1 in the Lin^−^ population by flow cytometry 1 week (A) and 3 weeks (B) later, and cell number of the LSK cell population was counted. Data are shown as mean ± SEM (n = 3–4) and are representative of two independent experiments.
(TIF)

S2 Fig LSK cells in the BM cells of *IL-27* Tg mice have enhanced ability to differentiate into neutrophils. (A) An increased number of LSK cells in *IL-27* Tg mice. BM and spleen cells of WT and *IL-27* Tg mice were analyzed for the expression of c-Kit and Sca-1 in the Lin^−^ population by flow cytometry, and cell number of the LSK cell population was counted. (B-D) Augmented potential of LSK cells in *IL-27* Tg mice to differentiate into neutrophils, but markedly reduced differentiation into pDCs and cDCs. LSK populations (3 × 10^3^) purified from BM cells of WT and *IL-27* Tg mice were stimulated with GM-CSF. After 10 days, stimulated cells were analyzed for the expression of MHC class II and CD11c, and cell number of mDC (MHC class II^+^CD11c^+^) was counted (B). LSK populations (5 × 10^3^) were also stimulated with Flt3L and TPO. Ten days later, stimulated cells were analyzed for the expression of Siglec H and PDCA1 in the CD11c^+^ population, and the cell numbers of pDC and cDC were counted (C). The LSK populations were also stimulated with IL-3 and SCF. Six days later, these stimulated cells were analyzed regarding their multipotency in differentiating to Ly6G^+^CD11b^+^ neutrophils, F4/80^+^CD11b^+^ macrophages, c-Kit^+^FcεR1α^+^CD11b^−^ mast cells, and CD49b^+^FcεR1α^+^CD11b^−^ basophils, and the cell numbers of respective cells were counted (D). Data are shown as mean ± SEM (n = 3) and are representative of two to four independent experiments. *P < 0.05, **P < 0.01, ***P < 0.005.
(TIF)

S3 Fig Increased expression of *Cebpb* in the LSK cells expanded by IL-27 and SCF, and the highest expression of *WSX-1* in primary LSK cells among hematopoietic progenitors. RNA was prepared from the LSK cells expanded by IL-27 and SCF for 2 weeks together with primary LSK cells and other progenitors, and subjected to real-time RT-PCR. Data are shown as mean ± SEM (n = 2–4) and are representative of two independent experiments.
(TIF)

S4 Fig IL-27 alone, but not SCF alone, induces phosphorylation of STAT1 and STAT3. Flow cytometry histogram analysis of primary LSK cells after stimulation with the combination with IL-27 (10 ng/ml) and SCF (10 ng/ml), IFN-γ (100 U/ml) and SCF (10 ng/ml), or each alone for 60 min using anti-pY-STAT1 or anti-pY-STAT3 (solid line) and control antibody (plain line with shading). Data are shown as mean ± SEM (n = 3).
(TIF)

S5 Fig Time course of parasitemia and serum IFN-γ level in WT and *WSX-1*-deficient mice after infection with the blood stage of *P*. *berghei* XAT. WT mice and *WSX-1*-deficient mice were infected with the blood stage of *P*. *berghei* XAT and parasitemia was measured with time course after infection (A). Serum IFN-γ level was determined 14 days later (B). Data are shown as mean ± SEM (n = 3–5) and are representative of at least two independent experiments. *P < 0.05, **P < 0.01.
(TIF)

S6 Fig Reduced potential of LSK cells from *WSX-1*-deficient mice to differentiate into myeloid cells compared with those from WT mice after malaria infection. LSK cells in the BM and spleen of WT and *WSX-1*-deficient mice infected with malaria for 7 days were purified and differentiated into myeloid cells *in vitro* by IL-3 and SCF, and cell number of differentiated cells was measured. Data are shown as mean ± SEM (n = 3) and are representative of at least two independent experiments. *P < 0.05, **P < 0.01, ***P < 0.005.
(TIF)

S7 Fig Promotion of expansion of LSK cells by IL-27 in a cell-autonomous direct manner. BM cells (1 × 10^6^) from CD45.1 congenic mice and BM cells (1 × 10^6^) from *WSX-1*-deficient mice (CD45.2) were equally mixed and transferred into lethally (9 Gy) irradiated CD45.2 recipient mice. After 7 days, these mice were infected with the blood stage of *P*. *berghei* XAT; an additional 7 days later, and populations of LSK cells (A) and neutrophils (B) in the BM and spleen were analyzed by flow cytometry. Representative dot plots of CD45.1^+^ and CD45.2^+^ cells in these populations are shown and percentages of these CD45.1^+^ and CD45.2^+^ cells in each population were compared. Data are shown as mean ± SEM (n = 3–4). *P < 0.05, ***P < 0.005.
(TIF)

S8 Fig Expression of cytokine and chemokine mRNA in the BM and spleen after malaria infection. WT and *IFN-γ*-deficient mice were infected with the blood stage of *P*. *berghei* XAT, and RNA was prepared from BM and spleen of WT and *IFN-γ*-deficient mice 7 days after the infection, and the mRNA expression levels of cytokines were analyzed as indicated by real-time RT-PCR. Data are shown as mean ± SEM (n = 3) and are representative of two independent experiments. *P < 0.05, ***P < 0.005.
(TIF)

S9 Fig Augmentation of mRNA expression of *IL-27 p28*, but not *IL-6*, by IFN-γ in WT BM cells *in vitro*. Total BM cells of WT mice were stimulated with IFN-γ (100 U/ml) for 48 hr and the mRNA expression of *IL-27 p28*, *EBI3*, and *IL-6* was analyzed by real-time RT-PCR (A). IL-27 p28 levels in culture supernatants were also determined by ELISA (B). Data are shown as mean ± SEM (n = 3) and are representative of two independent experiments. *P < 0.05, ***P < 0.005.
(TIF)

S10 Fig mRNA expression of transcription factors and molecule critical for cell differentiation and proliferation in *STAT1* KO and *STAT3* cKO LSK cells. (A) LSK cells purified from BM cells of WT (129) mice and *STAT1*-deficient mice were expanded by IL-27 and SCF for 2 weeks, and the LSK population was then purified by sorting and subjected to real-time RT-PCR. (B) Purified GFP^−^ *STAT3* ^*flox/flox*^ LSK cells and GFP^+^ *STAT3* cKO LSK cells were expanded by IL-27 and SCF for 10 days, and the LSK population was then purified by sorting and subjected to real-time RT-PCR. Data are shown as mean ± SEM (n = 3–4) and representative of two to three independent experiments. *P < 0.05, ***P < 0.005.
(TIF)

S11 Fig There was no apparent difference in the parasitemia and expansion of LSK cells and neutrophils between WT mice and *WSX-1*-deficient mice 7 days after lethal *P*. *berghei* NK65 infection. WT or *WSX-1*-deficient mice were infected with the blood stage of *P*. *berghei* NK65. Seven days later, parasitemia was determined (A), and populations of LSK cells (B) and neutrophils (C) in the BM and spleen were analyzed by flow cytometry, and representative dot plots of c-Kit and Sca-1 in the Lin^−^ population and CD11b and Gr-1 are shown. Cell number of these populations in the BM and spleen was also counted. Data are shown as mean ± SEM (n = 2–4). *P < 0.05.
(TIF)

S12 Fig Dispensable role of IL-6 in infection with the blood stage of *P*. *berghei* XAT. (A) Comparable susceptibility of WT and *IL-6*-deficient mice to malaria. WT and *IL-6*-deficient mice were infected with the blood stage of *P*. *berghei* XAT and parasitemia was counted at 7 days after infection. Data are shown as mean ± SEM (n = 5) and are representative of at least two independent experiments. *P < 0.05, **P < 0.01, ***P < 0.005. (B-C) Similar cell numbers of the LSK cell population and neutrophils in the BM and spleen of WT and *IL-6*-deficient mice compared with mice infected with malaria. BM and spleen cells were analyzed by flow cytometry 7 days after malaria infection; representative dot plots of c-Kit and Sca-1 in the Lin^−^ population (B) and Gr-1 and CD11b (C) are shown. The cell numbers of the LSK cell population and neutrophils were counted (B-C). Data are shown as mean ± SEM (n = 3) and are representative of two independent experiments. *P < 0.05, **P < 0.01, ***P < 0.005.
(TIF)
