# Supplementary figures and images for: Promotion of Expansion and Differentiation of Hematopoietic Stem Cells by Interleukin-27 into Myeloid Progenitors to Control Infection in Emergency Myelopoiesis
Source: PLoS Pathog. 2016 Mar 18;12(3):e1005507. doi: 10.1371/journal.ppat.1005507 (PMC4798290; doi:10.1371/journal.ppat.1005507)

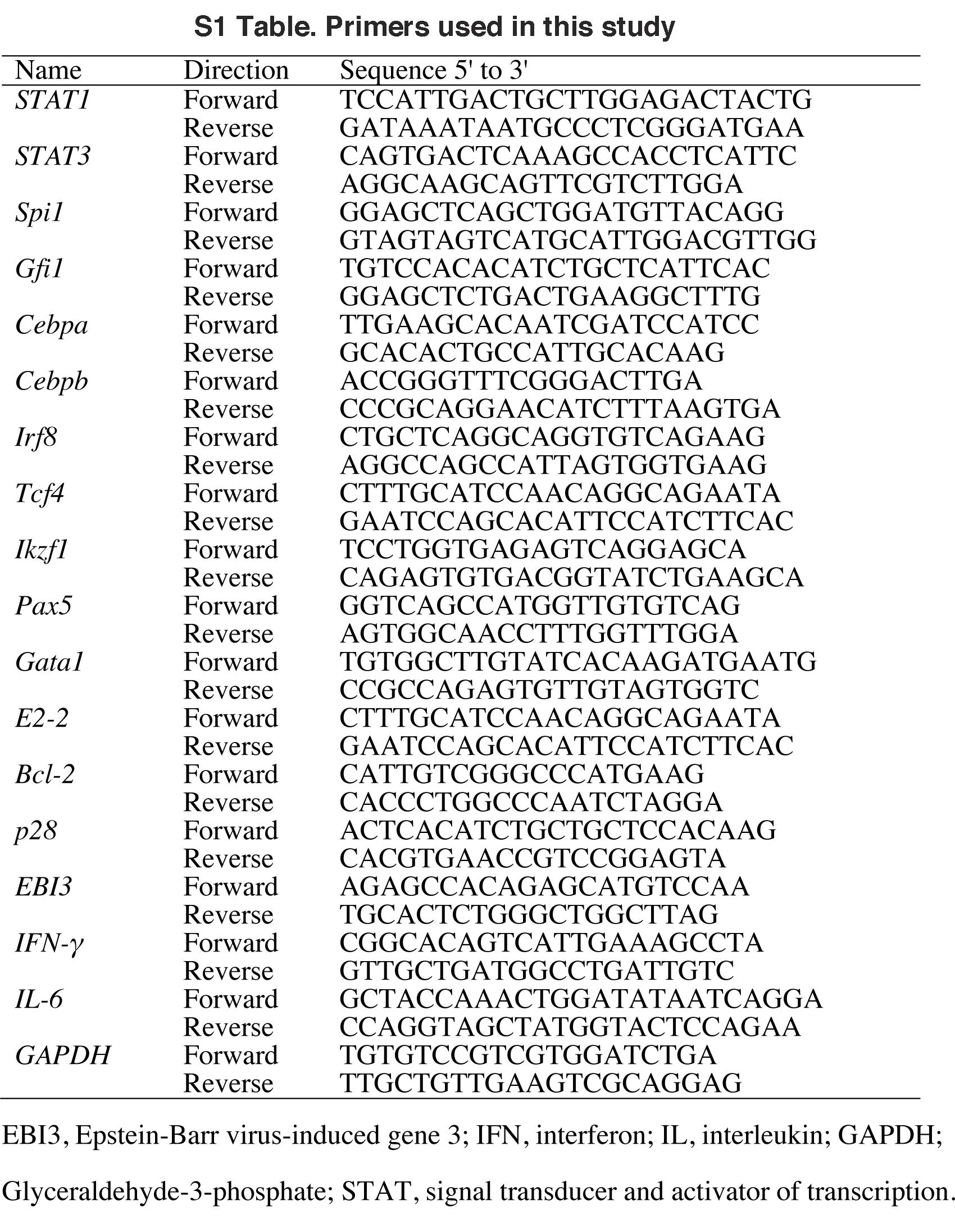

Supplement: S1 Table — (TIF) [file ppat.1005507.s001.tif]

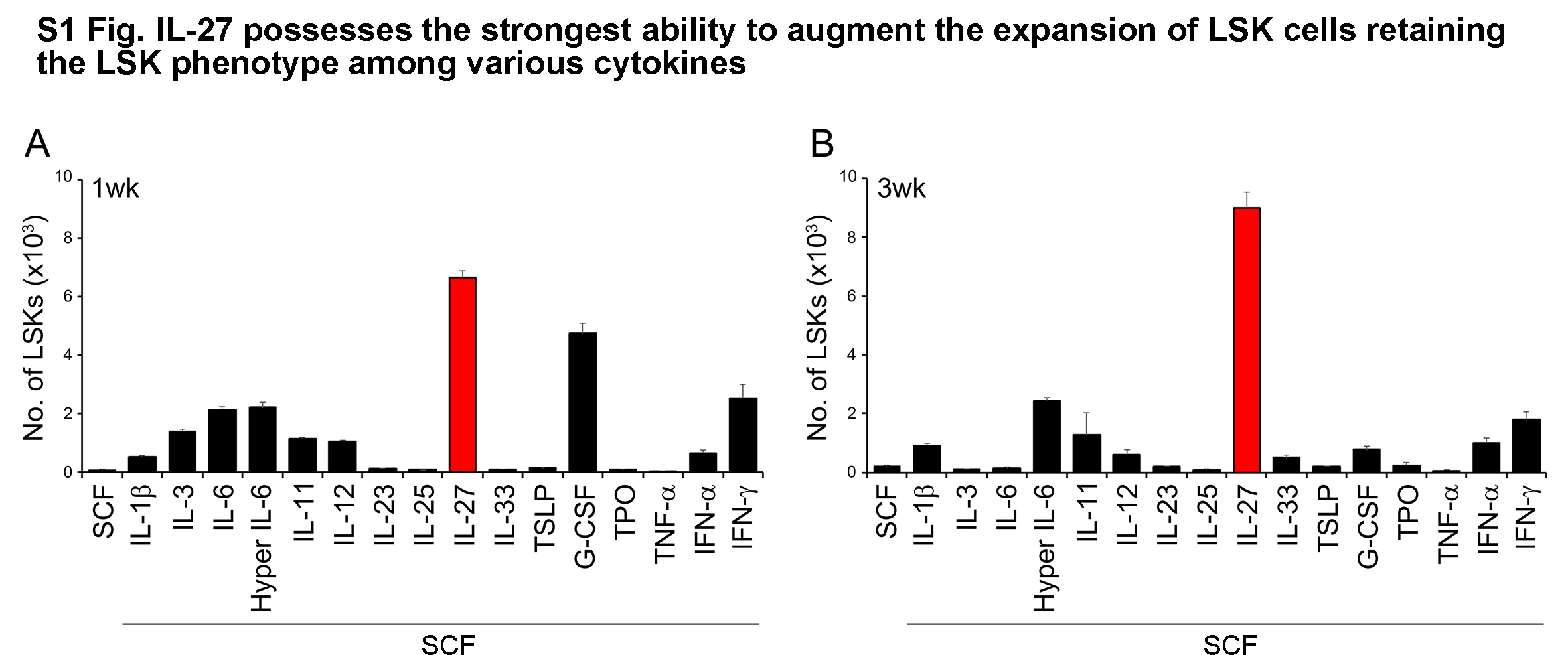

Supplement: S1 Fig — LSK cells (5 × 10^2^) from WT mice were stimulated by various cytokines together with SCF. The stimulated and expanded cells were analyzed for expression of c-Kit and Sca-1 in the Lin− population by flow cytometry 1 week (A) and 3 weeks (B) later, and cell number of the LSK cell population was counted. Data are shown as mean ± SEM (n = 3–4) and are representative of two independent experiments. (TIF) [file ppat.1005507.s002.tif]

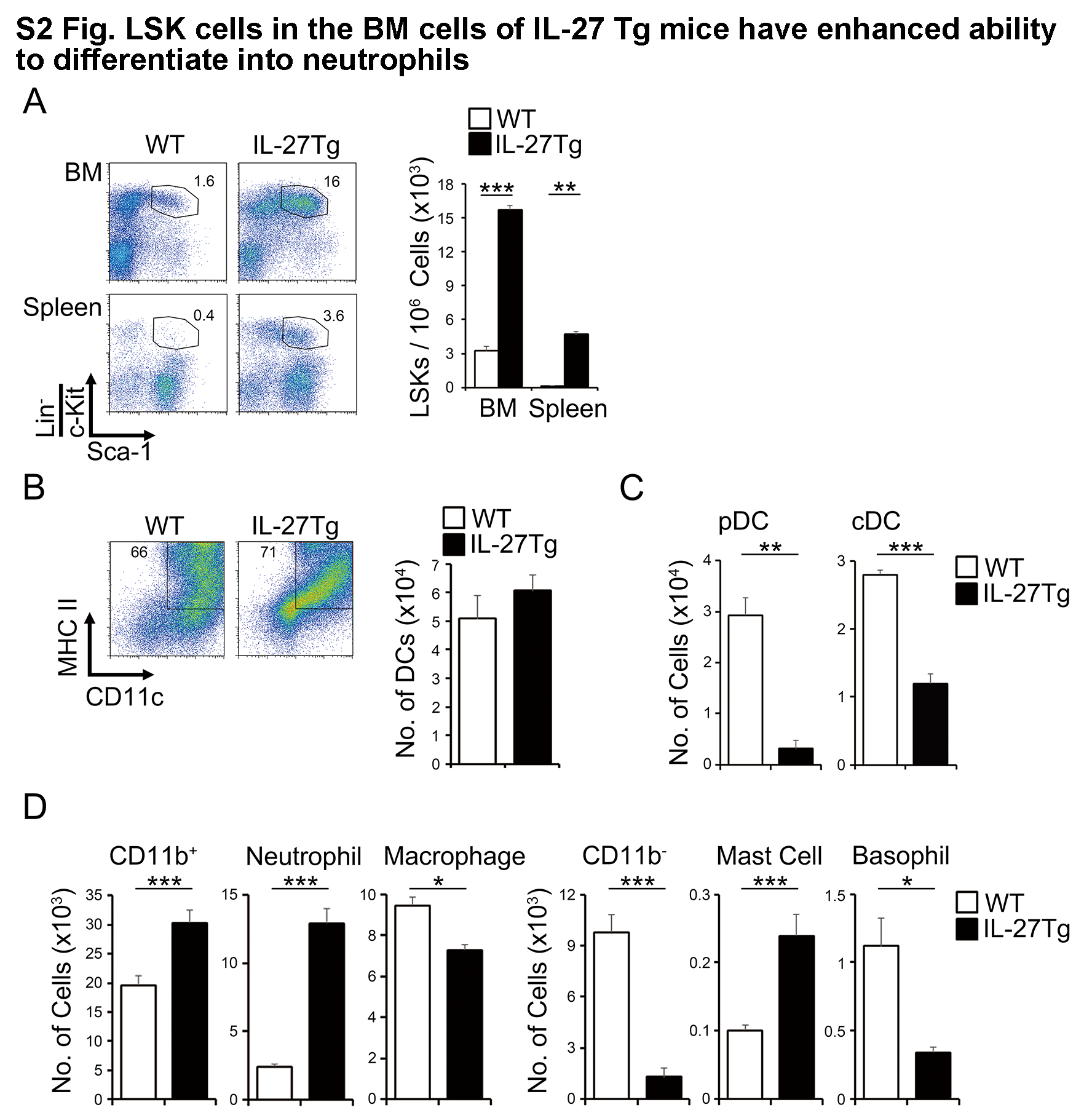

Supplement: S2 Fig — (A) An increased number of LSK cells in IL-27 Tg mice. BM and spleen cells of WT and IL-27 Tg mice were analyzed for the expression of c-Kit and Sca-1 in the Lin− population by flow cytometry, and cell number of the LSK cell population was counted. (B-D) Augmented potential of LSK cells in IL-27 Tg mice to differentiate into neutrophils, but markedly reduced differentiation into pDCs and cDCs. LSK populations (3 × 10^3^) purified from BM cells of WT and IL-27 Tg mice were stimulated with GM-CSF. After 10 days, stimulated cells were analyzed for the expression of MHC class II and CD11c, and cell number of mDC (MHC class II+CD11c+) was counted (B). LSK populations (5 × 10^3^) were also stimulated with Flt3L and TPO. Ten days later, stimulated cells were analyzed for the expression of Siglec H and PDCA1 in the CD11c+ population, and the cell numbers of pDC and cDC were counted (C). The LSK populations were also stimulated with IL-3 and SCF. Six days later, these stimulated cells were analyzed regarding their multipotency in differentiating to Ly6G+CD11b+ neutrophils, F4/80+CD11b+ macrophages, c-Kit+FcεR1α+CD11b− mast cells, and CD49b+FcεR1α+CD11b− basophils, and the cell numbers of respective cells were counted (D). Data are shown as mean ± SEM (n = 3) and are representative of two to four independent experiments. *P < 0.05, **P < 0.01, ***P < 0.005. (TIF) [file ppat.1005507.s003.tif]

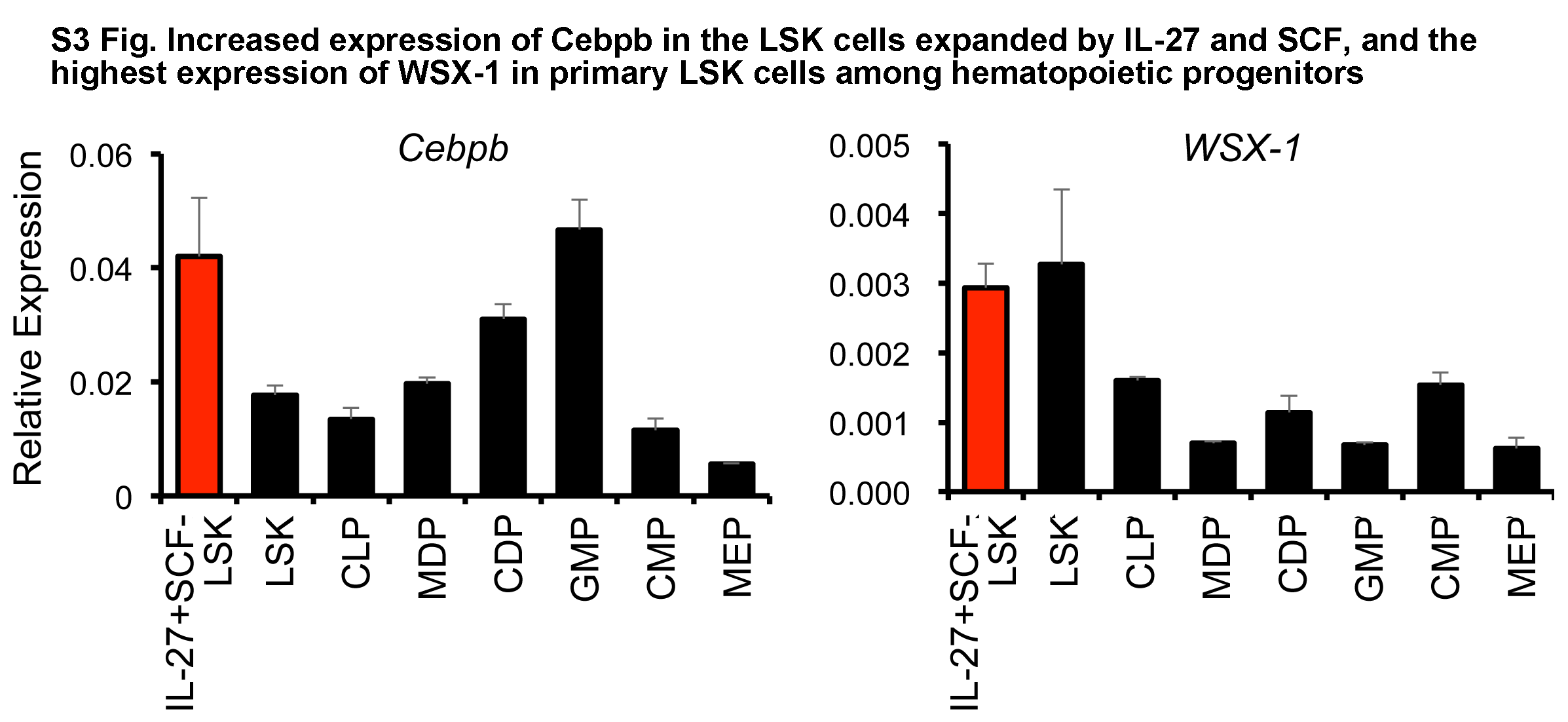

Supplement: S3 Fig — RNA was prepared from the LSK cells expanded by IL-27 and SCF for 2 weeks together with primary LSK cells and other progenitors, and subjected to real-time RT-PCR. Data are shown as mean ± SEM (n = 2–4) and are representative of two independent experiments. (TIF) [file ppat.1005507.s004.tif]

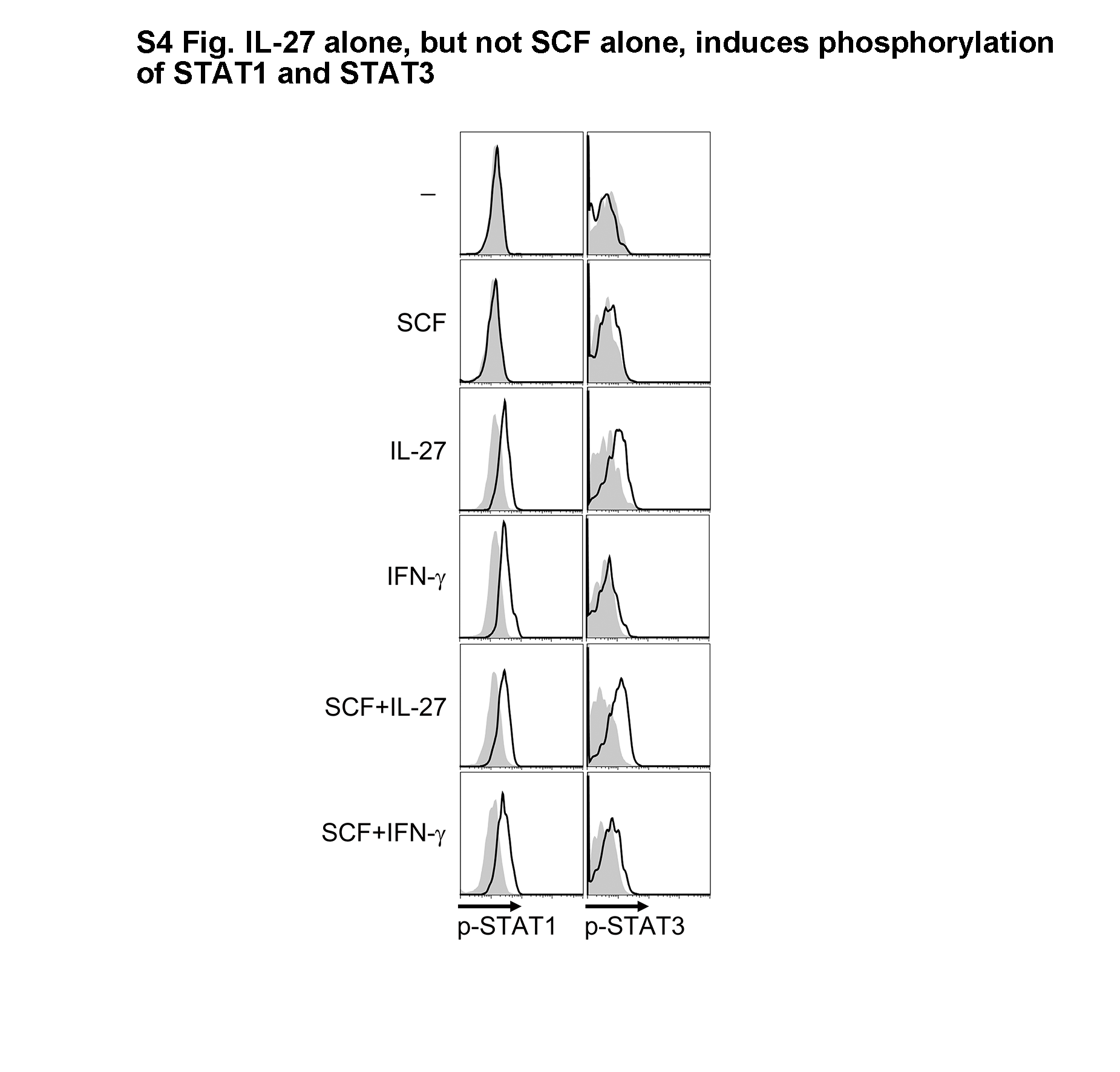

Supplement: S4 Fig — Flow cytometry histogram analysis of primary LSK cells after stimulation with the combination with IL-27 (10 ng/ml) and SCF (10 ng/ml), IFN-γ (100 U/ml) and SCF (10 ng/ml), or each alone for 60 min using anti-pY-STAT1 or anti-pY-STAT3 (solid line) and control antibody (plain line with shading). Data are shown as mean ± SEM (n = 3). (TIF) [file ppat.1005507.s005.tif]

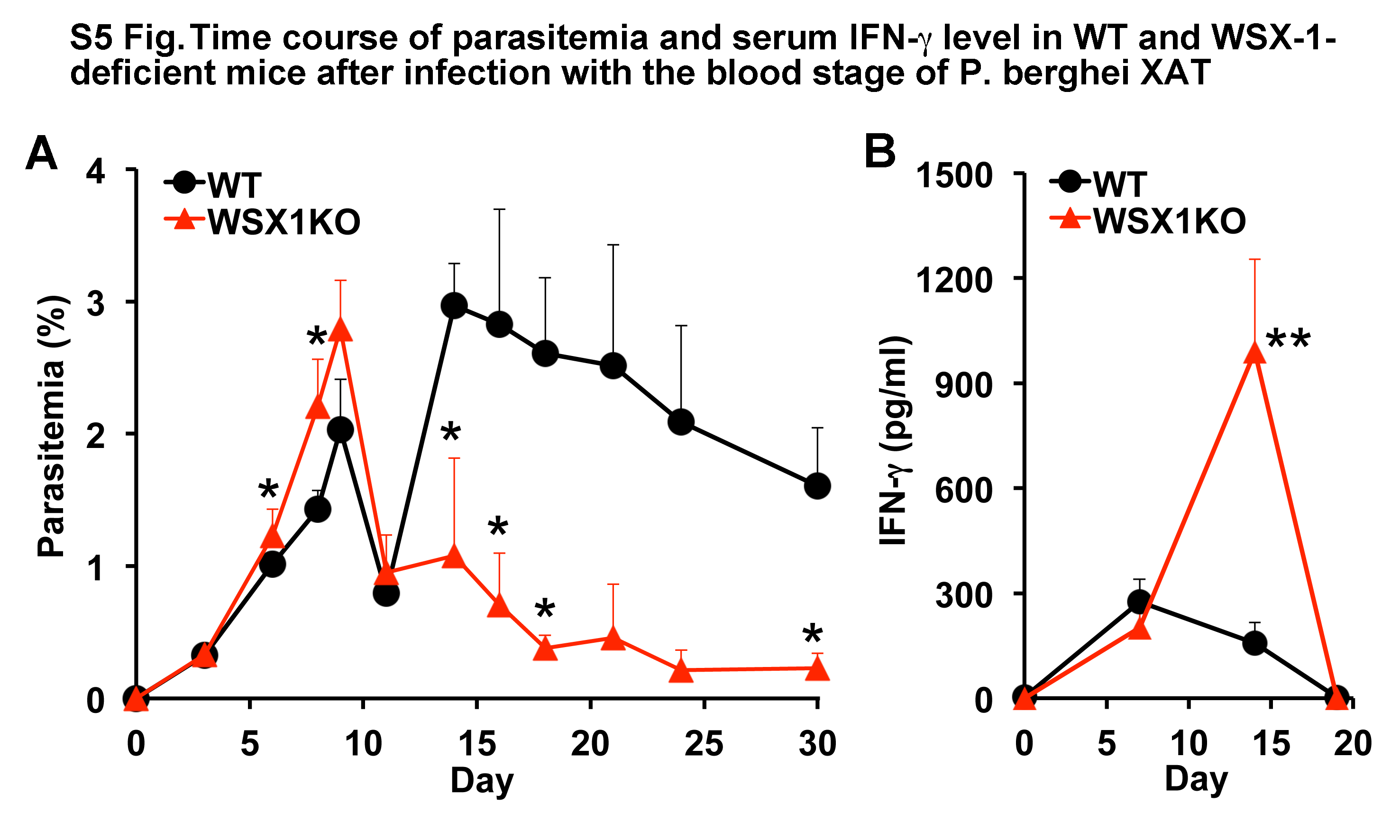

Supplement: S5 Fig — WT mice and WSX-1-deficient mice were infected with the blood stage of P. berghei XAT and parasitemia was measured with time course after infection (A). Serum IFN-γ level was determined 14 days later (B). Data are shown as mean ± SEM (n = 3–5) and are representative of at least two independent experiments. *P < 0.05, **P < 0.01. (TIF) [file ppat.1005507.s006.tif]

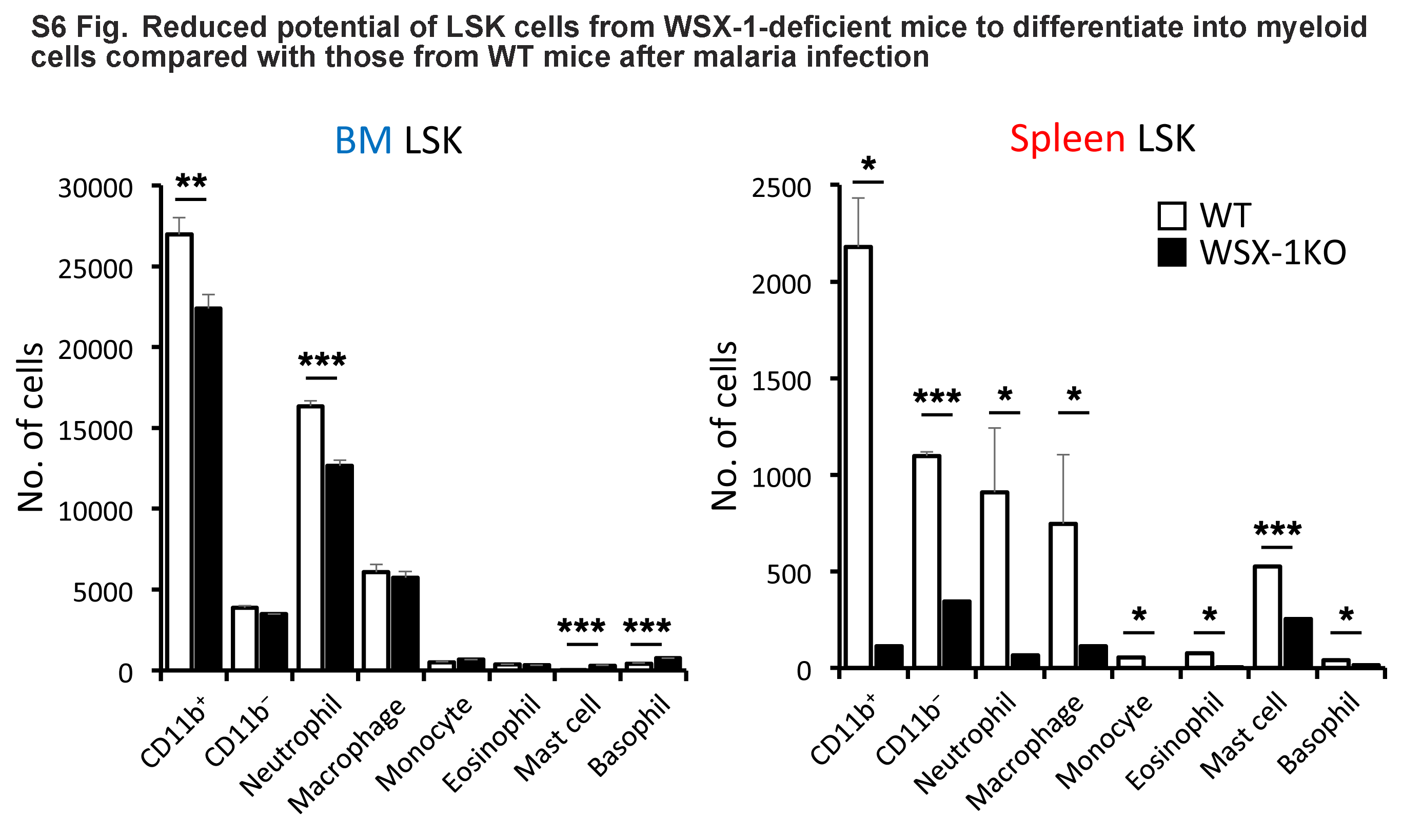

Supplement: S6 Fig — LSK cells in the BM and spleen of WT and WSX-1-deficient mice infected with malaria for 7 days were purified and differentiated into myeloid cells in vitro by IL-3 and SCF, and cell number of differentiated cells was measured. Data are shown as mean ± SEM (n = 3) and are representative of at least two independent experiments. *P < 0.05, **P < 0.01, ***P < 0.005. (TIF) [file ppat.1005507.s007.tif]

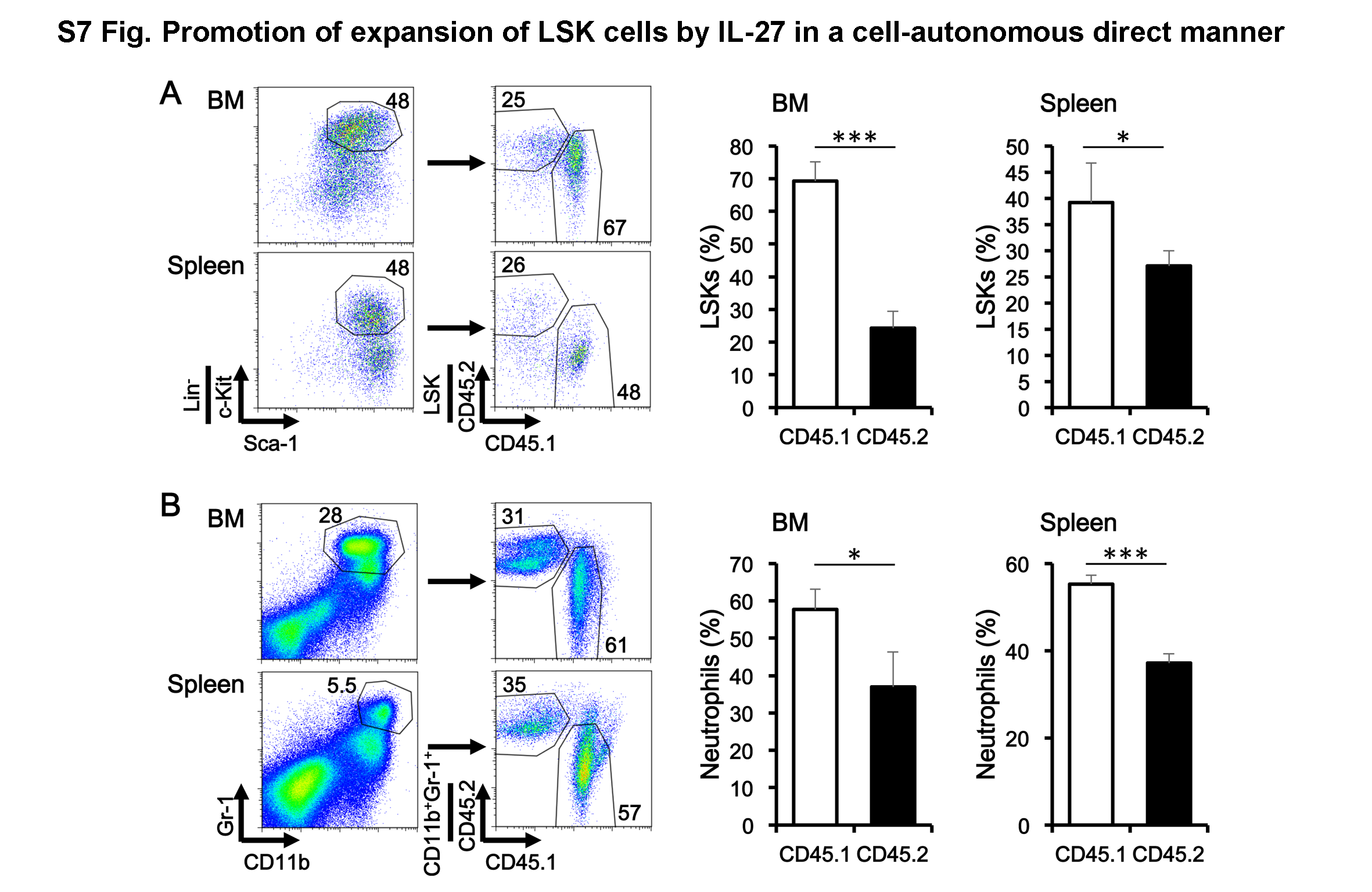

Supplement: S7 Fig — BM cells (1 × 10^6^) from CD45.1 congenic mice and BM cells (1 × 10^6^) from WSX-1-deficient mice (CD45.2) were equally mixed and transferred into lethally (9 Gy) irradiated CD45.2 recipient mice. After 7 days, these mice were infected with the blood stage of P. berghei XAT; an additional 7 days later, and populations of LSK cells (A) and neutrophils (B) in the BM and spleen were analyzed by flow cytometry. Representative dot plots of CD45.1+ and CD45.2+ cells in these populations are shown and percentages of these CD45.1+ and CD45.2+ cells in each population were compared. Data are shown as mean ± SEM (n = 3–4). *P < 0.05, ***P < 0.005. (TIF) [file ppat.1005507.s008.tif]

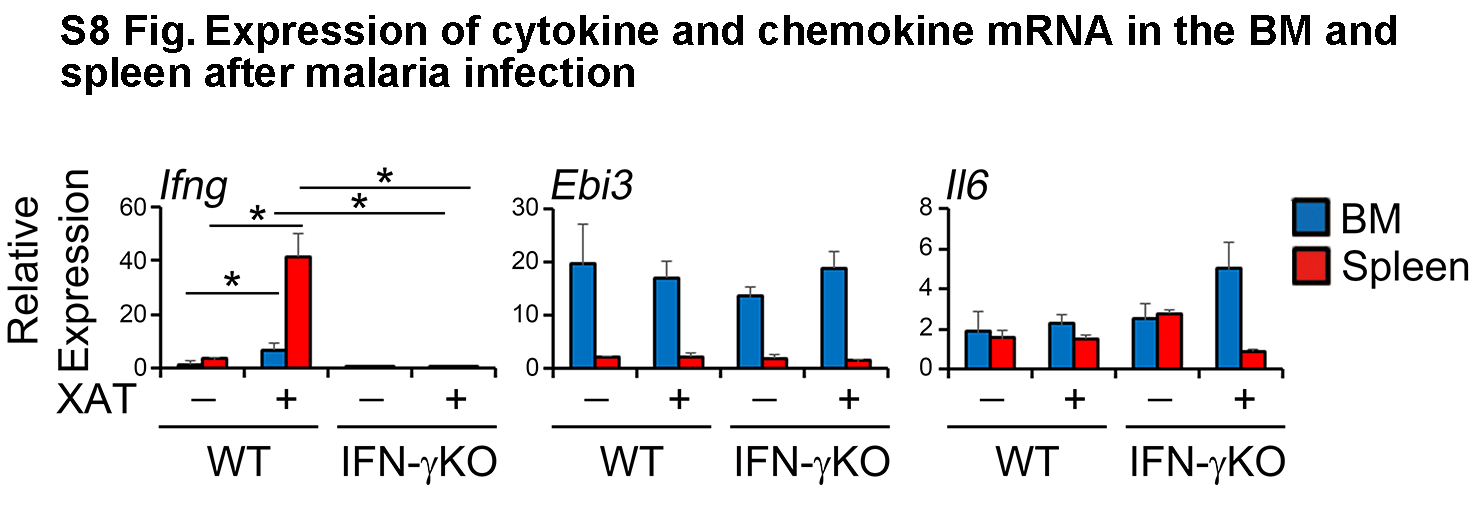

Supplement: S8 Fig — WT and IFN-γ-deficient mice were infected with the blood stage of P. berghei XAT, and RNA was prepared from BM and spleen of WT and IFN-γ-deficient mice 7 days after the infection, and the mRNA expression levels of cytokines were analyzed as indicated by real-time RT-PCR. Data are shown as mean ± SEM (n = 3) and are representative of two independent experiments. *P < 0.05, ***P < 0.005. (TIF) [file ppat.1005507.s009.tif]

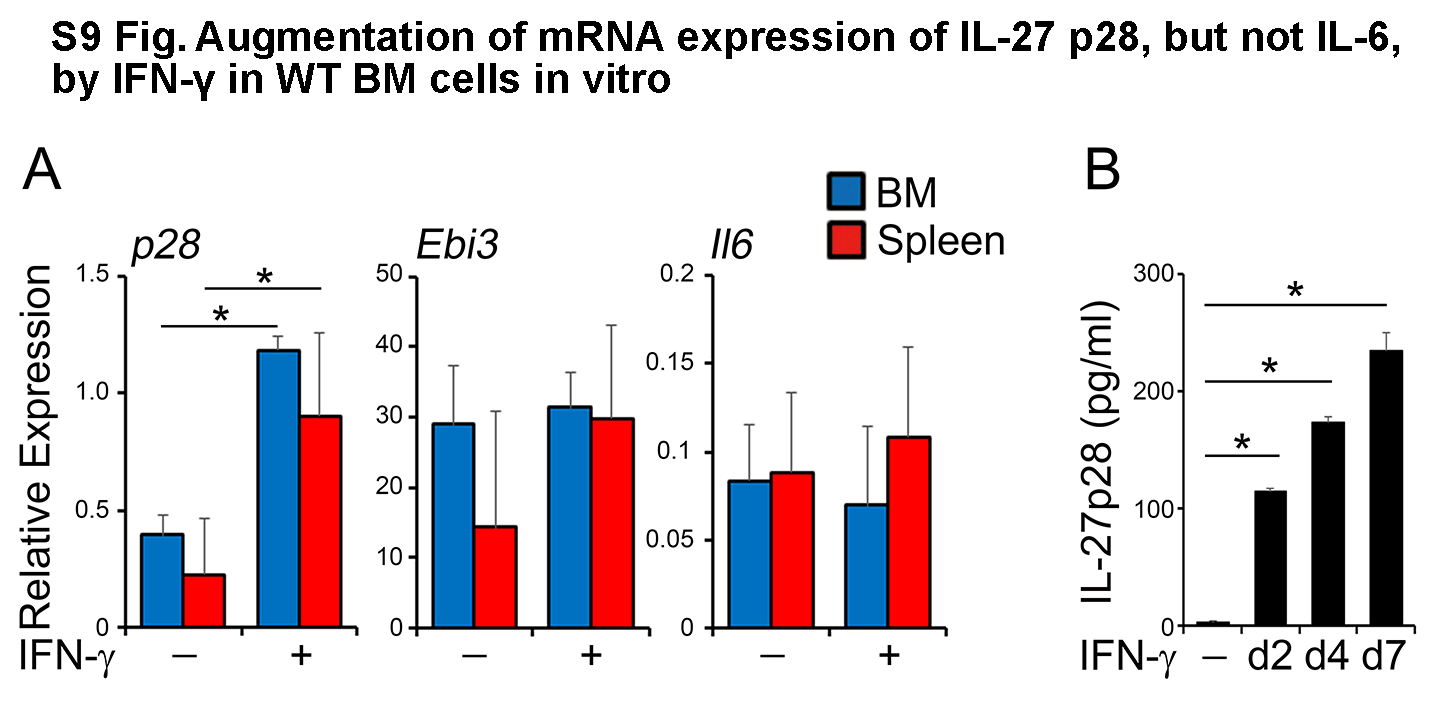

Supplement: S9 Fig — Total BM cells of WT mice were stimulated with IFN-γ (100 U/ml) for 48 hr and the mRNA expression of IL-27 p28, EBI3, and IL-6 was analyzed by real-time RT-PCR (A). IL-27 p28 levels in culture supernatants were also determined by ELISA (B). Data are shown as mean ± SEM (n = 3) and are representative of two independent experiments. *P < 0.05, ***P < 0.005. (TIF) [file ppat.1005507.s010.tif]

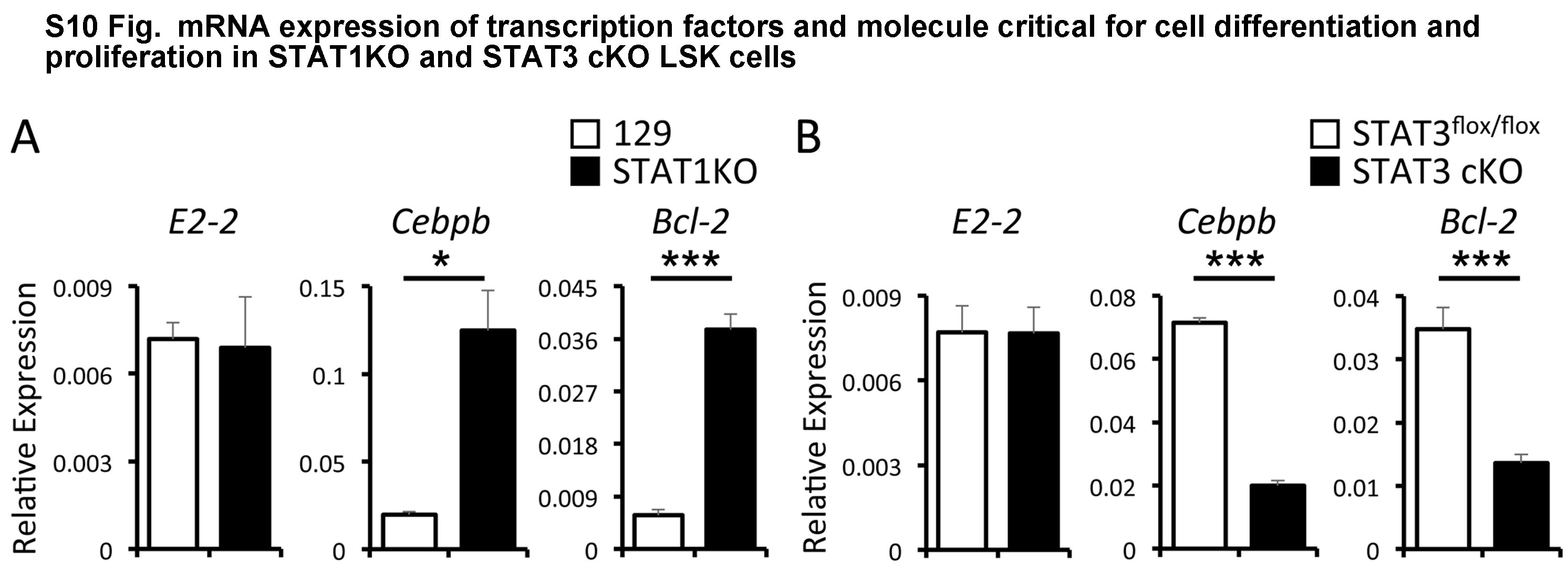

Supplement: S10 Fig — (A) LSK cells purified from BM cells of WT (129) mice and STAT1-deficient mice were expanded by IL-27 and SCF for 2 weeks, and the LSK population was then purified by sorting and subjected to real-time RT-PCR. (B) Purified GFP− STAT3 flox/flox LSK cells and GFP+ STAT3 cKO LSK cells were expanded by IL-27 and SCF for 10 days, and the LSK population was then purified by sorting and subjected to real-time RT-PCR. Data are shown as mean ± SEM (n = 3–4) and representative of two to three independent experiments. *P < 0.05, ***P < 0.005. (TIF) [file ppat.1005507.s011.tif]

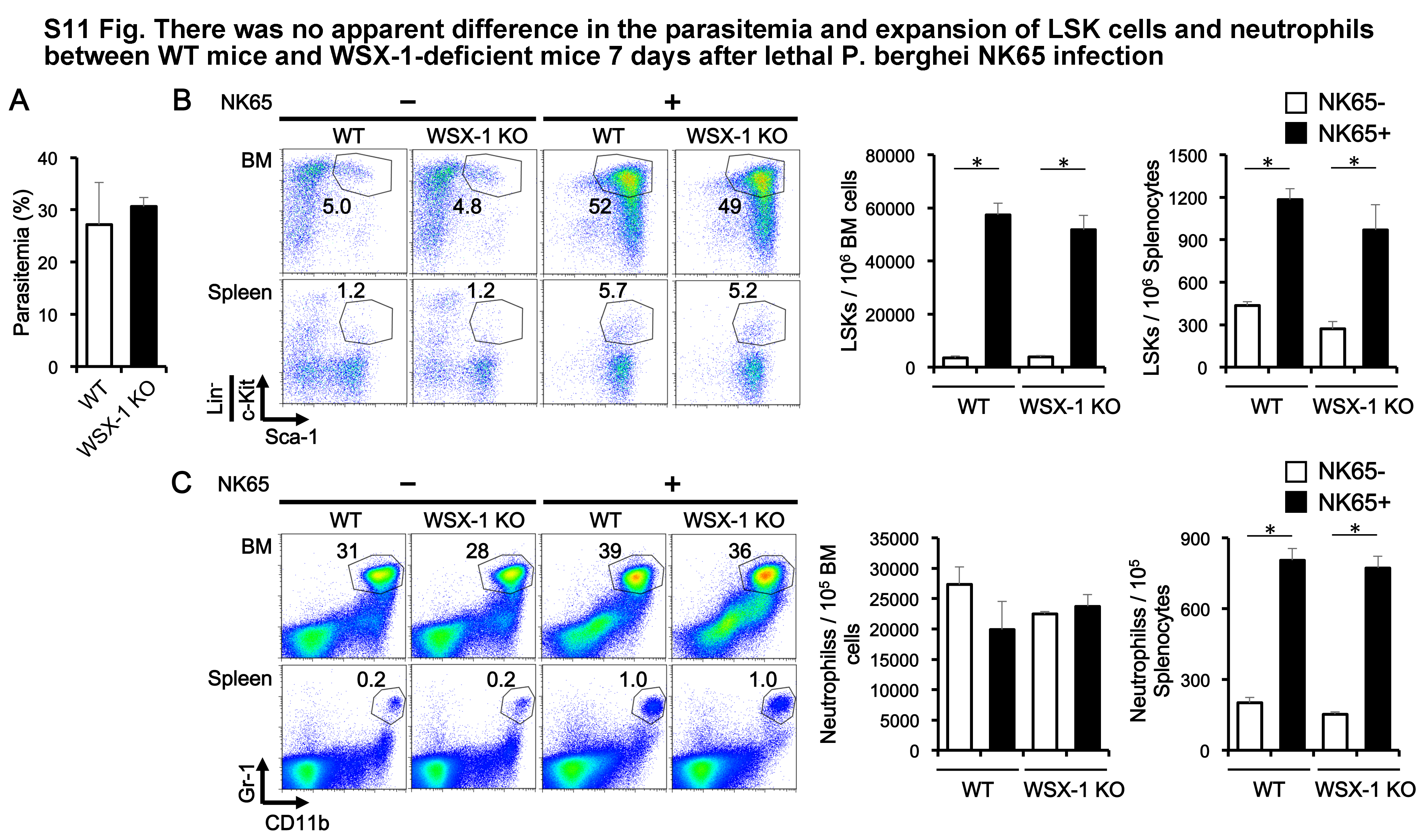

Supplement: S11 Fig — WT or WSX-1-deficient mice were infected with the blood stage of P. berghei NK65. Seven days later, parasitemia was determined (A), and populations of LSK cells (B) and neutrophils (C) in the BM and spleen were analyzed by flow cytometry, and representative dot plots of c-Kit and Sca-1 in the Lin− population and CD11b and Gr-1 are shown. Cell number of these populations in the BM and spleen was also counted. Data are shown as mean ± SEM (n = 2–4). *P < 0.05. (TIF) [file ppat.1005507.s012.tif]

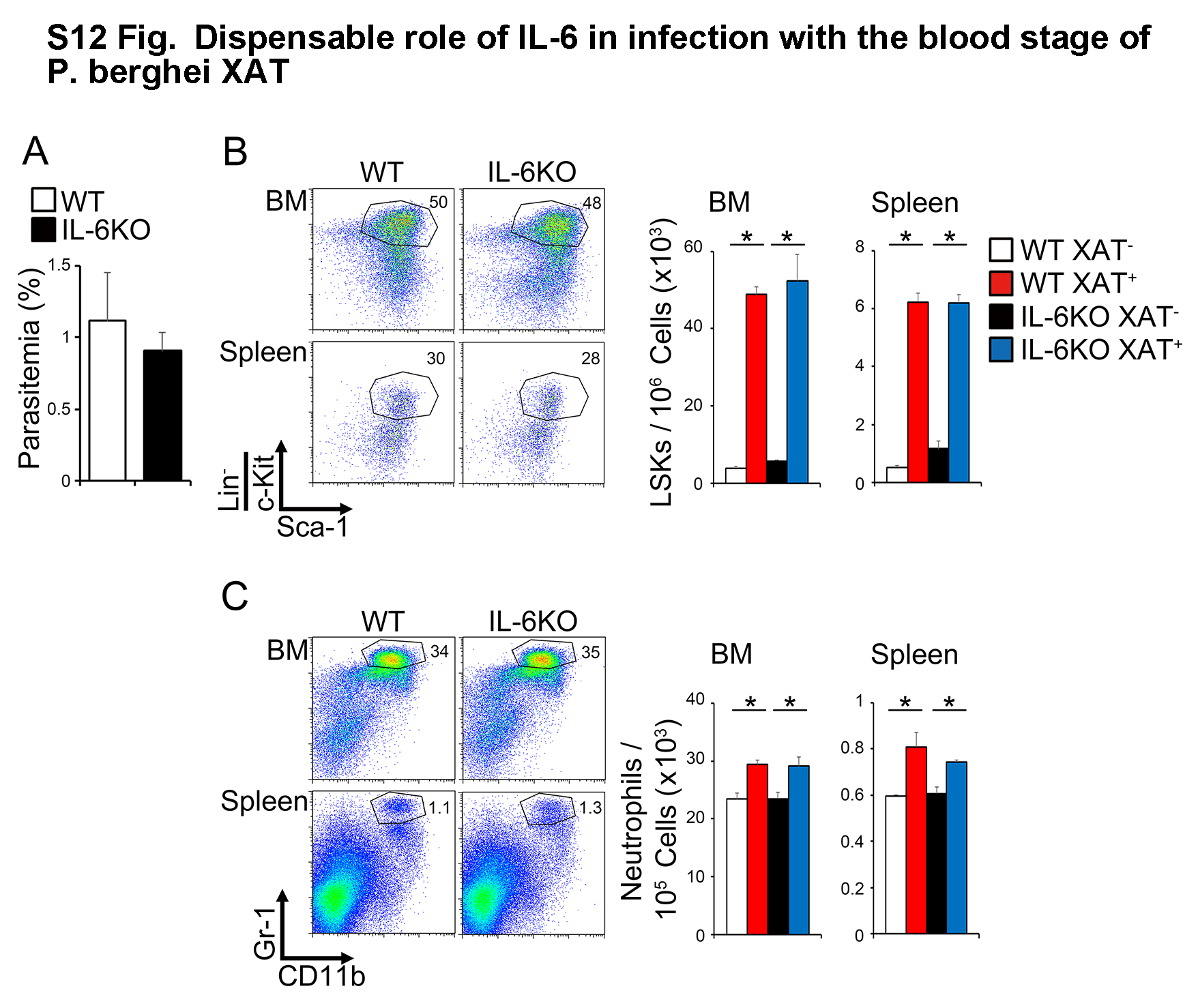

Supplement: S12 Fig — (A) Comparable susceptibility of WT and IL-6-deficient mice to malaria. WT and IL-6-deficient mice were infected with the blood stage of P. berghei XAT and parasitemia was counted at 7 days after infection. Data are shown as mean ± SEM (n = 5) and are representative of at least two independent experiments. *P < 0.05, **P < 0.01, ***P < 0.005. (B-C) Similar cell numbers of the LSK cell population and neutrophils in the BM and spleen of WT and IL-6-deficient mice compared with mice infected with malaria. BM and spleen cells were analyzed by flow cytometry 7 days after malaria infection; representative dot plots of c-Kit and Sca-1 in the Lin− population (B) and Gr-1 and CD11b (C) are shown. The cell numbers of the LSK cell population and neutrophils were counted (B-C). Data are shown as mean ± SEM (n = 3) and are representative of two independent experiments. *P < 0.05, **P < 0.01, ***P < 0.005. (TIF) [file ppat.1005507.s013.tif]
